# *O*-glycosylation of the extracellular domain of pollen class I formins modulates their plasma membrane mobility

**DOI:** 10.1093/jxb/erac131

**Published:** 2022-04-06

**Authors:** Cecilia M Lara-Mondragón, Alexandria Dorchak, Cora A MacAlister

**Affiliations:** Department of Molecular, Cellular and Developmental Biology, University of Michigan, Ann Arbor, MI, USA; Department of Molecular, Cellular and Developmental Biology, University of Michigan, Ann Arbor, MI, USA; Department of Molecular, Cellular and Developmental Biology, University of Michigan, Ann Arbor, MI, USA; Ohio State University, USA

**Keywords:** Actin, cell wall, cytoskeleton, formin, glycosylation, pollen tube, tip growth

## Abstract

In plant cells, linkage between the cytoskeleton, plasma membrane, and cell wall is crucial for maintaining cell shape. In highly polarized pollen tubes, this coordination is especially important to allow rapid tip growth and successful fertilization. Class I formins contain cytoplasmic actin-nucleating formin homology domains as well as a proline-rich extracellular domain and are candidate coordination factors. Here, using Arabidopsis, we investigated the functional significance of the extracellular domain of two pollen-expressed class I formins: AtFH3, which does not have a polar localization, and AtFH5, which is limited to the growing tip region. We show that the extracellular domain of both is necessary for their function, and identify distinct *O*-glycans attached to these sequences, AtFH5 being hydroxyproline-arabinosylated and AtFH3 carrying arabinogalactan chains. Loss of hydroxyproline arabinosylation altered the plasma membrane localization of AtFH5 and disrupted actin cytoskeleton organization. Moreover, we show that *O*-glycans differentially affect lateral mobility in the plasma membrane. Together, our results support a model of protein sub-functionalization in which AtFH5 and AtFH3, restricted to specific plasma membrane domains by their extracellular domains and the glycans attached to them, organize distinct subarrays of actin during pollen tube elongation.

## Teaser

In Arabidopsis pollen tubes, class I formins, transmembrane proteins, organize subarrays of actin during pollen tube elongation and show lateral mobility and localization dependent on *O*-glycosylation of their extracellular domain.

## Introduction

Plant cells are enclosed in a polysaccharide-rich extracellular matrix, the cell wall. Interconnection between the cytoskeleton, plasma membrane, and cell wall is crucial in shaping plant cells, during cell growth, and in response to stimuli (Baluška *et al.*, 2003; Jaillais and Ott, 2020; Chebli *et al.*, 2021). In highly polarized plant cells, such as pollen tubes, the coordination of the F-actin cytoskeleton, secretion machinery, and cell wall assembly is pivotal to allow fast growth (Bascom *et al.*, 2018). Furthermore, cytoskeleton and cell wall coordination permit timely delivery and proper positioning of plasma membrane or plasma membrane-associated proteins, allowing the pollen tube to respond to mechanical and chemical cues along its journey through the pistil (Chebli *et al.*, 2012; Dresselhaus and Franklin-Tong, 2013; Hafidh and Honys, 2021). In pollen tubes, as in other plant cells, the precise mechanism by which cell wall, plasma membrane, and cytoskeleton establish a linkage is not fully understood, although it is believed that interactions between these structures vary depending on the tissue and cell type (Chebli *et al.*, 2021). Evidence of interaction between a GPI-anchored proteoglycan, ARABINOXYLAN PECTIN ARABINOGALACTAN PROTEIN1 (APAP1), and pectic polysaccharides in the wall was reported previously (Tan *et al.*, 2013), further suggesting that covalent interactions between distinct biomolecules in the cell wall exist.

Class I formins are transmembrane proteins with a Pro-rich extracellular domain (ECD) and intracellular actin-nucleating Formin Homology (FH1 and FH2) domains (van Gisbergen and Bezanilla, 2013), making them suitable candidates to mediate cell wall–plasma membrane–cytoskeleton linkage. While the actin nucleating/bundling activity of members of this family of proteins in Arabidopsis has been thoroughly studied (Blanchoin and Staiger, 2010; Wang *et al.*, 2012), our understanding of the functional significance of the ECD is limited to a handful of reports. The ECD of class I formins generally possess a high content of Pro residues, resembling the glycosylation motifs of hydroxyproline-rich cell wall glycoproteins (HRGPs). HRGP-like motifs present in the ECDs of class I formins belong to two subgroups: extensins (EXT) and arabinogalactan glycoproteins (AGP) (Borassi *et al.*, 2016; Liu *et al.*, 2016). EXTs are highly repetitive proteins defined by the presence of Ser-Pro_(3–5)_ motifs (Kieliszewski and Lamport, 1994). Proline residues are enzymatically converted to hydroxyproline (Hyp) and then modified by the addition of short linear chains of arabinosides (Shpak *et al.*, 2001; Petersen *et al.*, 2021). AGPs, on the other hand, are glycosylated by the addition of branched arabinogalactan (AG) glycans in their clustered dipeptide Ser-Pro, Thr-Pro, Ala-Pro, and Gly-Pro repeats (Tan *et al.*, 2003). In Arabidopsis, the vegetative formin AtFH1 is immobilized in the plasma membrane by the interaction of its ECD EXT-like motifs and the cell wall (Martinière *et al.*, 2011). Similarly, it was reported that the interaction between the ECD of SYMBIOTIC FORMIN 1 (SYFO1) and the cell wall is necessary to induce root hair curling during nodule development in *Medicago truncatula* (Liang *et al.*, 2021). Both reports highlight the importance and versatility of the ECD in formin cell wall anchoring; however, further studies are necessary to elucidate the nature of this interaction. Despite the evidence of the importance of the EXT-like motifs present in the ECD of class I formins and their putative role in protein immobilization, experimental evidence of *O*-glycosylation of such motifs is lacking.

Two members of the class I formin family, AtFH3 and AtFH5, regulate cortical actin polymerization during pollen germination and tube elongation (Ye *et al.*, 2009; Cheung *et al.*, 2010; Liu *et al*., 2018, 2021). Genetically tagged versions of AtFH3 and AtFH5 showed distinct localization patterns in pollen tubes: AtFH3 localizes throughout the pollen tube’s plasma membrane, while AtFH5 is restricted to the apical plasma membrane. Based on their localization patterns and genetic studies (Cheung *et al.*, 2010; Lan *et al.*, 2018), it is hypothesized that AtFH3 and AtFH5 participate in the organization of distinct subarrays of actin microfilaments. AtFH3 stimulates the polymerization and bundling of actin filaments in the pollen tube shank (Ye *et al.*, 2009), whereas AtFH5 mediates the assembly of a fine network of apical and subapical actin (Cheung *et al.*, 2010). The mechanistic basis of class I formin sub-functionalization during pollen tube elongation remains to be described; however, the observation that the replacement of the ECD of AtFH5 with the intracellular FH1/2 domains of AtFH3 mimics the localization of wild-type AtFH5 (Lan *et al.*, 2018) suggests that the ECD might be responsible for their spatial patterning and important for their functional diversification.

Here, we further explored the functional significance of the ECD of pollen class I formins AtFH3 and AtFH5. We demonstrate that the ECD of both AtFH3 and AtFH5 is necessary for their plasma membrane localization. Furthermore, we provide evidence that the HRGP-like motifs in their ECDs are post-translationally modified by the addition of distinct *O*-glycans, consistent with predictions based on the Hyp-contiguity hypothesis. Additionally, our results suggest that these post-translational modifications likely modulate their interaction with the extracellular matrix and lateral mobility in the plasma membrane.

## Materials and methods

### Plant material and growth conditions

Arabidopsis plants were grown under long day photoperiods (16 h light and 8 h dark) in a temperature-controlled growth room at 23 °C. The *hpat1-2* (SALK_120066), *hpat2-2* (SM_3_38225), and *hpat3-1* (SALK_047668) triple mutant’s recovery was described previously (MacAlister *et al.*, 2016). *fh3-1* (SALK_150350) and *fh5-2* (SALK_044464) T-DNA insertion alleles in the Columbia-0 background (Lan *et al.*, 2018) were obtained from the Arabidopsis Biological Resource Center (ABRC). *fh3-1* and *fh5-2* lines were genotyped with the primers published by Lan *et al.* (2018). *fh3-1* or *fh5-2* was crossed with the *hpat1,2,3* mutant to generate higher order mutants *hpat1,2,3/fh3-1* and *hpat1,2,3/fh5-2*. The primers used for genotyping of the *hpat1,2,3* triple mutant are listed in Supplementary Table S1.

### Molecular cloning and plant transformation

The coding region of AtFH1 (AT3G25500.1), AtFH3 (AT4G15200.1), and AtFH5 (AT5G54650.1) was amplified from cDNA derived from leaves (AtFH1) or pollen (AtFH3/5) using the Phusion® High-Fidelity DNA Polymerase (M0530S, NEB) and the primers are listed in Supplementary Table S1. ECD and modified versions were generated by overlap extension PCR. PCR fragments were cloned into Gateway entry vectors using BP Clonase II (11789-020; Thermo Fisher Scientific). Then full length and ECD modified versions were recombined using LR Clonase II into a modified pFAST-R01 binary vector (Shimada *et al.*, 2010). Cloning of mNeonGreen into the pFAST-R01 was performed as in Beuder *et al.* (2020) for protein localization. For photoconversion assays, mEosFP was cloned into the same vector, pFAST-R01. For protein purification and fluorescence recovery after photobleaching (FRAP) assays, the signal peptide, ECD, and transmembrane domain of AtFH1, AtFH3, and AtFH5 were amplified from entry clones using the primers listed in Supplementary Table S1, adding attB recombination sites for Gateway cloning. The PCR products were recombined using LR Clonase II into the pMDC83 binary vector for CaMV 35S promoter expression (Curtis and Grossniklaus, 2003). Arabidopsis plants were transformed by the floral dipping method (Clough and Bent, 1998).

### Pollen assays

Pollen germination medium (PGM) modified from (Rodriguez-Enriquez *et al.*, 2013) (10% (w/v) sucrose, 0.01% (w/v) boric acid, 1 mM CaCl_2_, 1 mM Ca(NO_3_)_2_, 1 mM KCl, 0.03% (w/v) casein enzymatic hydrolysate, 0.01% (w/v) *myo*-inositol, 0.1 mM spermidine, 10 mM γ‐aminobutyric acid, 500 μM methyl jasmonate, pH adjusted to 8.0, and for solid PGM, solidified with 1% (w/v) low melting temperature agarose) was used for all *in vitro* growth assays and live-cell imaging. For pollen live-cell imaging, CoverWell (Grace Bio-Labs, GBL635051) silicone chambers were placed on top of a glass slide, filled with molten PGM and solidified on a flat surface for ~1 min. Once solidified, pollen grains were dusted on top of the medium and carefully covered with a coverslip. The samples were incubated for 50 min at room temperature in a humid chamber consisting of a plastic box with damp paper towels prior to imaging.

### Live-cell imaging

Pollen tubes expressing the full length and altered ECD versions of AtFH3 and AtFH5 fused to mNG from three independent transgenic lines were imaged using a Leica SP5 laser scanning confocal microscope, with a 488 nm excitation laser, an RSP500 dichroic beam splitter and HyD detectors capturing signal in the 588–670 nm wavelength range. *z*-Stacks were captured throughout the width of each pollen tube, with automatically optimized *z*-slice steps. At least 15 pollen tubes were imaged for each construct in both Columbia and *hpat1,2,3* backgrounds. Image analysis was performed using ImageJ. To measure fluorescence intensity, a segmented line along the pollen tube periphery, starting from the tip pole towards the shank, was drawn in the medial *z*-section. Using the *plot profile* tool in ImageJ, the pixel gray value along the line distance was measured, with distance 0 representing the tip pole. The values of fluorescence intensity over distance for each of the genotype–construct combinations were fitted using a linear mixed-effect model with a random slope accounting for within-group variability in cell fluorescence (see Supplementary Table S2) using the *lme4* package in R (Bates *et al.*, 2015). A maximum likelihood ratio test was used to determine the best fit model, and results of this test and *lme4* diagnostics are shown in Supplementary Table S2. Coefficient estimates were extracted and compared for statistical significance using the *sJPlot* R package.

For FM4-64 staining and brefeldin A (BFA) treatment, pollen tubes expressing AtFH3:mNG or AtFH5:mNG in the Columbia and *hpat1,2,3* background were grown as described under ‘Pollen assays’, but incubated without coverslips. After 45 min of incubation, 12 µM of FM4-64 in liquid PGM was added on top of the pollen tubes and incubated for an additional 15 min (total incubation 60 min). Pollen tubes were then incubated for 60 min with the mock treatment (PGM+methanol) or BFA treatment (25 µM BFA in PGM). For plasmolysis experiments, pollen tubes expressing AtFH3:mNG or AtFH5:mNG in the Columbia background were grown as mentioned above, and after 45 min of incubation on regular solid PGM, they were transferred to imaging chambers with solid PGM with 25% of sucrose to induce plasmolysis; 12 µM FM4-64 in liquid PGM with 25% sucrose was added on top and pollen tubes were then incubated for 10 min. A coverslip was placed gently on top of the silicone chamber, and samples imaged using a Leica SP5 laser scanning confocal microscope with the same settings as above for mNG, and for FM4-64 laser excitation was set up at 514 nm wavelength with a DD458/514 dichroic beam splitter, and a HyD detector capturing light in a 620–783 nm wavelength range was used.

FRAP assays were performed in epidermal cells of Arabidopsis stable lines expressing AtFH1ecd:GFP, AtFH3ecd:GFP, or AtFH5ecd:GFP, using the FRAP LAS application wizard of the Leica TCS SP8 confocal microscope. For excitation, the white light laser was set up for excitation at 488 nm, the notch filter set to NF488, and the PMT detector captured light in the 496–558 nm wavelength range, maintaining minimum laser intensity to prevent unwanted photobleaching during time-lapse imaging. Photobleaching of a circular region of interest (ROI; 1 µm^2^ area) in a single *z*-plane was performed using the white light laser set up at 488 nm and 100% intensity for 10 s. Recovery of green fluorescent protein (GFP) fluorescence was documented by capturing images every 2 s for 1 min post-photobleaching. For mEosFP photoconversion assays in pollen tubes expressing AtFH3:mEosFP, AtFH5:mEosFP, or deletion versions of their respective Pro-rich regions (AtFH3Δ[P]:mEosFP or AtFH5Δ[P]:mEosFP), with excitation of the green from of mEosFP (mEosFP-G), the white light laser was set up at 505 nm, while for the red form of mEosFP (mEosFP-R) it was set up at 569 nm; the notch filter was set at NF488/561/633, and PMT detectors captured light at 490–516 nm wavelength for mEosFP-G and 570–635 nm for mEosFP-R. Photoconversion of an ROI (for AtFH3 a rectangular ROI of 8 µm^2^ area in the pollen tube’s subapical region or shank, for AtFH5 a circular ROI of 5 µm^2^ area near the subapical region of the pollen tube) was achieved with a 405 laser diode, at 80% intensity for 8–14 s in the pollen tube medial plane. After photoconversion, recovery of mEosFP-G was recorded for 1–2 min. Recovery curves for GFP were calculated according to Zheng *et al.* (2011). Kymographic analyses of the mEosFP photoconversion data were performed by measuring the fluorescence intensity along the pollen tube periphery over time, and then the mean fluorescence values were normalized to their maximum value and kymographs built in RStudio.

### Pollen tube F-actin immunolabeling

F-actin staining of pollen tubes was performed following the protocol of Qu *et al.* (2020) with some modifications. Briefly, Columbia wild-type and *hpat1,2,3* pollen tubes were grown on a pad of solid PGM pH 7 for 50 min in a humid chamber at room temperature (three biological replicates, *n*=20 pollen tubes per replicate). To disrupt HRGP *O*-glycosylation or perturb AGP function, wild-type pollen tubes were treated with 30 µM β-Yariv or increasing concentrations of 3-4-dehydro-DL-proline (3-4-DHP, 10, 20, or 30 µM) dissolved in liquid PGM and incubated for 45 min prior to the fixation step (*n*=10 pollen tubes per treatment, two biological replicates). Additionally, to determine the effect of the deletion of FH1/FH2 domains on actin organization, wild-type pollen tubes, *fh3-1*, *fh5-2*, and *fh3-1* AtFH3ΔECD:mNG or *fh5-2* AtFH5ΔECD:mNG lines were grown in the same system (imaging chambers with sold PGM pH 7 for 50 min, *n*>15 pollen tubes per genotype, two biological replicates). After incubation, pollen tubes were incubated for 1 h at 28 °C with fixative (300 µM *m*-maleimidobenzoyl-*N*-hydroxysulfosuccinimide ester (MBS) in liquid PGM pH 7). The fixative was removed by capillarity using Kimwipes (Kimtech, AA120) and the tubes were washed for 10 min with wash buffer 1 (150 µM MBS in liquid PGM pH 7, 0.05% v/v Nonidet P-40), followed by three washes for 10 min each with wash buffer 2 (50 mM Tris–HCl pH 7.4, 200 mM NaCl, 10% (w/v) sucrose, 0.05% (v/v) Nonidet P-40). Pollen tubes were then incubated with CytoPainter Phalloidin-iFluor 488 reagent (1:1000 in wash buffer 2; Abcam, ab176753) or CytoPainter Phalloidin-iFluor 594 (1:1000 in wash buffer 2; Abcam, ab176757) overnight at 4 °C in the dark. The following day, pollen tubes were washed twice with wash buffer 2 for 10 min, protecting samples from light. A coverslip was placed carefully on top of the PGM pad and the samples were imaged with the Leica SP5 laser scanning confocal microscope, using a 488 nm excitation laser, an RSP500 dichroic beam splitter, and HyD detectors capturing signal in the 495–600 nm wavelength range for samples incubated with CytoPainter Phalloidin-iFluor 488, while samples incubated with CytoPainter Phalloidin-iFluor 594 were imaged using a 561 nm excitation laser, and DD458/514 dichroic beam splitter and HyD detectors capturing light in the 590–670 nm wavelength range. All genotypes were imaged using identical settings (laser intensity, gain and line averaging). *z*-Stacks were taken throughout the width of each pollen tube, with a *z*-slice step of 0.5 µm. Image analysis was performed using ImageJ. Signal intensity was measured in maximum intensity projections as the mean gray value in a 5 µm×10 µm rectangular region in the pollen tube tip. To measure filament angles, semi-projections of two to three adjacent *z*-slices were generated and filament angle degree with respect to the axis of growth was measured. From 20 pollen tubes of each genotype, *n*≥700 filament angles were measured, and their respective distributions plotted. 3D surface plots were built using the orthogonal view and 3D interactive surface plot tools in ImageJ.

### Genetic complementation

Single insertion, Columbia wild-type lines expressing AtFH3:mNG or AtFH5:mNG and their ΔECD:mNG versions were introgressed to the *fh3-1* or the *fh5-2* background, respectively. Germination percentage was measured after 3 h of incubation for the wild-type, *fh3-1*, *fh5-2* mutant backgrounds and complemented lines *fh3-1C* or *fh3-1* AtFH3ΔECD:mNG and *fh5-2C* or *fh5-2* AtFH5ΔECD:mNG in three independent assays (*n*>1000).

### Microsome isolation, β-Yariv precipitation, and protein purification

Adult leaves from Arabidopsis stable lines expressing AtFH1ecd:GFP6×His, AtFH3ecd:GFP6×His and AtFH5ecd:GFP6×His or free GFP6×His under the CaMV 35S promoter were used as source for all protein assays. Protein methods are described in detail in Lara-Mondragón and MacAlister (2020). Briefly, ground frozen leaves were homogenized in extraction buffer (100 mM Tris–HCl pH 7.5, 8% sucrose, 5% glycerol, 10 mM EDTA, 10 mM EGTA, 5 mM KCl, 1 mM DTT, 1 mM PMSF, 0.4% casein and Pierce protease inhibitor cocktail (Thermo Fisher Scientific, A32953). The homogenate was incubated for 10 min with 4 mg of polyvinylpolypyrrolidone on ice, spun down for 3 min at 600 *g* and 4 °C to remove insoluble particles. The homogenate was filtered through a 70 μm pore size nylon strainer to remove tissue debris (Thermo Fisher Scientific, 22363548). Microsomal fractions were collected by ultracentrifugation at 100 000 *g* for 1 h at 4 °C. The microsomal fractions were emulsified in a buffer containing 10 mM Tris pH 7.5, 150 mM NaCl, 5 mM EDTA and 1% Triton X-100. Protein concentration of emulsified microsomes was determined using the Pierce BCA assay kit (Thermo Fisher Scientific, 23225).

Precipitation of AGPs was performed by incubating 1–2 mg of microsomal fractions with 1 mg ml^−1^ β-Yariv dissolved in 1% (w/v) NaCl overnight. The following day, samples were spun down at 21 000 *g* for 10 min and the pellet washed with 1% NaCl twice. Finally, the β-Yariv–AGP complex was dissociated by serially adding 250 μl of DMSO, 750 μl cold acetone and 10 μl of 2% NaCl and spun down at 21 000 *g* for 10 min. This last step was repeated two more times, and finally the pellet containing AGP glycoproteins was resuspended in sample buffer and separated in a 10% SDS-PAGE gel. The resolved fractions were then analysed by Western blot with anti-GFP antibody (Thermo Fisher Scientific A-11122).

### Phylogenetic analysis

Protein sequences of all 11 members of the class I formin family were aligned using ClustalX. Then, a maximum parsimony tree with 1000 bootstrap replicates was constructed using Phylip 3.698 (Felsenstein, 1989).

## Results

### The extracellular domain of pollen-expressed formins is necessary for plasma membrane localization

**Fig. 1.**
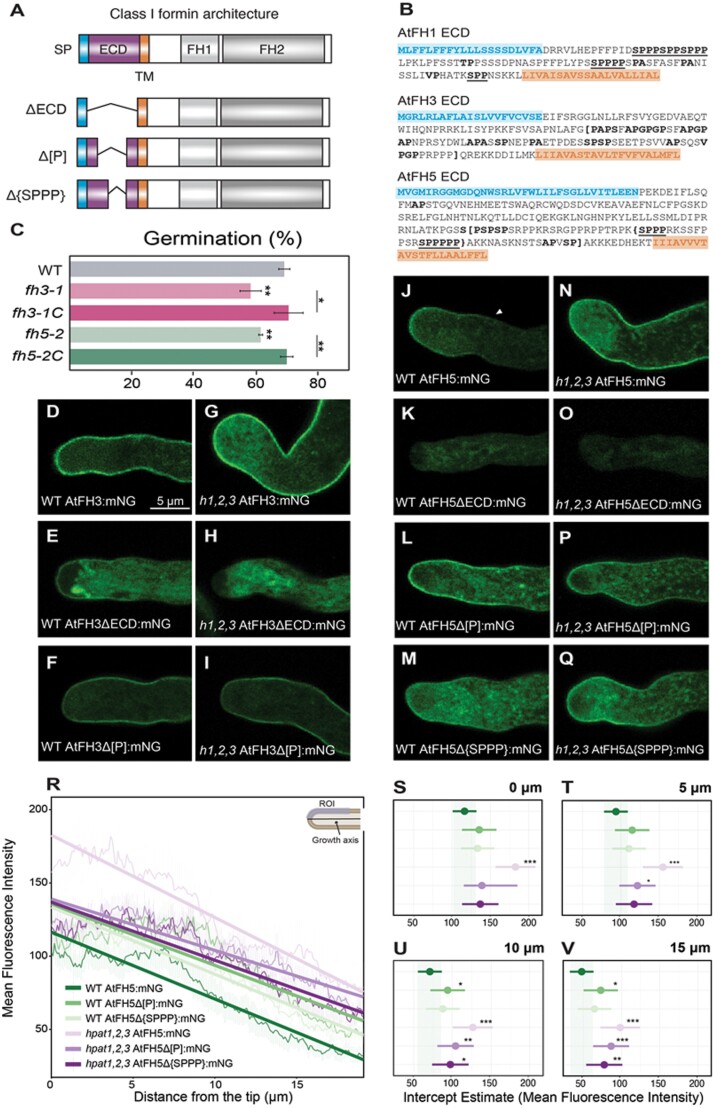
The loss of *O*-arabinosylation disrupts the localization of AtFH5. (A) General protein architecture of class I formins. ECD, extracellular domain; FH1, Formin Homology 1 domain; FH2, Formin Homology 2 domain; SP, signal peptide; TM, transmembrane domain. The altered versions of the ECD of AtFH3/5 are shown below. ΔECD, the entire extracellular domain was removed (purple region). In the Δ[P] versions, the Pro-rich region within square brackets (shown in (B)) was removed. Only for AtFH5, the Δ{SPPP} version lacked the EXT-like motifs within the brackets (AtFH5 ECD sequence, shown in (B)). (B) ECD sequences of AtFH1 (vegetative) and pollen expressed AtFH3 and AtFH5. Amino acids in blue correspond to the signal sequence (SP in (A)); residues in orange correspond to the transmembrane domain (TM in (A)); amino acids in bold correspond to predicted AG glycomodules, while bold underlined residues correspond to EXT-like glycosylation motifs. In AtFH3 and AtFH5 ECDs, regions within square or curly brackets correspond to deletions. (C) Germination defects of the *fh3-1* and *fh5-2* transcriptional null alleles are rescued by expression of the full length AtFH3 or AtFH5 fused to mNeonGreen (mNG). Pollen germination *in vitro* was measured after 3 h. Three biological replicates per genotype (*n*>1000); *P_adjusted_<0.05, **P_adjusted_<0.005: statistically significant differences (Student’s *t*-test). (D–I) Full length AtFH3, ΔECD, and Δ[P] versions expressed in WT (D–F) or *hpat1,2,3* (*h1,2,3*) (G–I) *in vitro* grown pollen tubes. (J–Q) Full length AtFH5, ΔECD, Δ[P] and Δ{SPPP} mNG fusions expressed in the WT background (J–M) or the *hpat1,2,3* background (N–Q). Arrowhead in (J) indicates the boundary of AtFH5 plasma membrane localization. (R) Linear mixed-effect model with random slope fitted to the data (fluorescence intensity over distance) measured for each construct–genotype combination. Mean fluorescence is shown in the plot as solid lines and shaded area represents standard error. Trendlines predicted by the model are shown in the same color scheme. (S–V) *y*-intercepts (mean fluorescence intensity) predicted by the model (circles) and their 95% confidence intervals (bars) at the tip (0 μm, S), subapical region (5 μm, T), 10 μm (U) or 15 μm (V) from the tip. *P<0.05, **P<0.005, ***P<0.001: statistically significant difference using the WT AtFH5:mNG as reference. *P*-values were derived from LMEM. Over 15 pollen tubes from three independent transgenic lines were measured per genotype and construct.

To evaluate the contribution of the ECD to the localization patterns of AtFH3 and AtFH5, we generated a series of C-terminus translational fusions of AtFH3 and AtFH5 to the fluorescent protein mNeonGreen (mNG) (Fig. 1A, B). Consistent with previous reports (Cheung *et al.*, 2010; Lan *et al.*, 2018), when expressed in the wild-type background, the localization of AtFH3 and AtFH5 displayed two distinct patterns: AtFH3 localized throughout the pollen tube periphery (Fig. 1D), whereas AtFH5 was restricted to the apical plasma membrane (Fig. 1J). Loss of function alleles of AtFH3 (*fh3-1*) or AtFH5 (*fh5-2*) display reduced pollen germination and pollen tube growth *in vitro* (Lan *et al.*, 2018). In our growth conditions, however, only pollen germination had a statistically significant reduction (Fig. 1C; see Supplementary Fig. S1A). We therefore only used pollen germination for further phenotypic analysis. To test whether our translational fusions were functional, we introgressed AtFH3:mNG into the *fh3-1* background and AtFH5:mNG into the *fh5-2* background, which resulted in full rescue of the germination defect, indicating that the fusion proteins were functional (Fig. 1C).

Interestingly, deletion of the ECD in both AtFH3 (Fig. 1E) and AtFH5 (Fig. 1K) resulted in a drastic reduction of the plasma membrane localization of the fusion proteins and intracellular accumulation, despite their possessing intact secretion signals and transmembrane domains (Fig. 1A). Introgression of AtFH3ΔECD:mNG and AtFH5ΔECD:mNG into the corresponding mutant backgrounds failed to rescue the germination defect, suggesting that the ECD of both AtFH3 and AtFH5 is necessary for their function (Supplementary Fig. S1B). Actin organization in the *fh3-1* and *fh5-2* mutant backgrounds was reported to be altered (Lan *et al.*, 2018), particularly with reduced actin accumulation and filament disorganization in the apical area. Since the deletion of the ECDs of AtFH3 and AtFH5 was unable to rescue the germination defect in their respective mutant backgrounds, we investigated whether actin organization remained defective in these lines. Consistent with prior observations by Lan *et al.* (2018), both *fh3-1* and *fh5-2* pollen tubes exhibited reduced actin labeling intensity in the apex compared with wild-type pollen tubes (Supplementary Fig. S2A, C) and, similarly, reduced accumulation of apical actin filaments was observed in the lines expressing the ΔECD:mNG versions of AtFH3 and AtFH5 (Supplementary Fig. S2D, E). Together, these results complement our genetic studies (Supplementary Fig. S1B) and suggest that the FH1/FH2 domains of AtFH3/5 are unable to function properly in the absence of their respective ECDs.

### The lack of hydroxyproline-*O*-arabinosylation alters AtFH5 patterning

Our results indicate that the ECD of class I formins is necessary for their proper plasma membrane localization (Fig. 1; Supplementary Fig. S1). The ECDs of both AtFH3 and AtFH5 are Pro-rich; AtFH3 possess clustered AGP-like glycosylation motifs, while AtFH5 contains EXT-like motifs (Fig. 1B). We hypothesized that the Pro-rich region of the ECD of these proteins, containing glycosylation motifs, might contribute to their distinct localization patterns. To explore this hypothesis, we investigated the effect of smaller deletions of the Pro-rich regions of AtFH3 (AtFH3Δ[P]:mNG) and AtFH5 (AtFH5Δ[P]:mNG) on their respective subcellular localization, and additionally, for AtFH5, a smaller deletion including its EXT-like motifs (AtFH5Δ{SPPP}:mNG) (Fig. 1A, B). The plasma membrane localization of AtFH3Δ[P]:mNG was unaffected compared with AtFH3:mNG (Fig. 1D, F). AtFH5:mNG exhibited a polar localization, restricted to the elongating tip (Fig. 1J). Interestingly, a moderate but significant expansion was observed in the localization of the version where the Pro-rich region of AtFH5 was deleted (AtFH5∆[P]:mNG), compared with the full-length version (Fig. 1J, L). To assess whether such differences in polarized patterning were statistically significant, we applied a linear mixed-effect model (LMEM) to the data, comprising measurements of the mean fluorescence intensity over distance, with 0 µm being the pollen tube tip (Fig. 1R; see Supplementary Table S2 for model parameters). The model shows a negative relationship between fluorescence intensity and distance (Fig. 1R; Supplementary Table S2): the mean fluorescence intensity (MFI) estimate for AtFH5:mNG in the wild-type background was 116.36 (95% CI: 100.92, 131.79) and decreased over distance reaching an estimate of 48.01 (95% CI: 32.61, 63.41) at 15 µm from the tip. AtFH5Δ[P]:mNG and AtFH5Δ{SPPP}:mNG were localized in the apical plasma membrane (Fig. 1L, M), although, the localization of the Pro-rich region deletion exhibited a significant increase in the MFI estimate at 10 µm (MFI=93.77 (95% CI: 71.42, 116.13), *P*<0.05) and at 15 µm from the tip in the AtFH5Δ[P]:mNG (MFI=72.94 (95% CI: 50.56, 95.31), *P*<0.05, Fig. 1V) version compared with AtFH5:mNG, suggesting that its plasma membrane localization extended beyond the subapical region. No significant differences in the intercept estimates of AtFH5Δ{SPPP}:mNG compared with the full-length AtFH5:mNG were observed.

Based on the localization analyses and altered localization observed in AtFH5Δ[P]:mNG, which contains HRGP-like motifs (Fig. 1B, R–V), we hypothesized that potential glycosylation of these residues is necessary for the apical localization of AtFH5. Hydroxyproline-*O*-arabinosyltransferases (HPATs) initiate the addition of arabinosides to Hyp residues within Ser-Pro_(3–5)_ motifs (Ogawa-Ohnishi *et al.*, 2013). The loss of function of HPAT1 and HPAT3 produces a severe male reproductive defect; pollen tubes lacking Hyp-*O*-arabinosylation (Hyp-Ara) display a range of phenotypes including reduced rates of elongation, initiation of secondary tips, and pollen tube rupture (MacAlister *et al.*, 2016; Beuder *et al.*, 2020). To further explore the effect of Hyp-*O*-arabinosylation in the localization of AtFH5, we expressed all previous constructs in the *hpat1,2,3* background and investigated changes in their subcellular localization. Interestingly, AtFH5:mNG greatly expanded its localization beyond the apical plasma membrane (Fig. 1J, N), supporting our hypothesis. LMEM intercept estimates for the full length AtFH5:mNG the *hpat1,2,3* background exhibited significant differences throughout the measured distances (Fig. 1S–V), starting at the tip with an estimated MFI of 182.64 (95% CI: 156.73, 208.56), *P*<0.001) and reaching at 15 µm an MFI estimate of 126.44 (95% CI: 100.61, 152.26), *P*<0.001). When expressed in the *hpat1,2,3* background, the localization AtFH5Δ[P]:mNG exhibited no difference in its MFI estimate at the tip or subapical area but a significant increase compared with wild-type farther from the tip (Fig. 1P, U,V); AtFH5Δ{SPPP}:mNG displayed a similar behavior, but a higher MFI estimate was only observed at 15 µm from the tip (Fig. 1Q, V). The deletion of the ECD for both AtFH3 and AtFH5, as in the WT background, caused intracellular accumulation in *hpat1,2,3* pollen tubes (Fig. 1H, O).

### Pollen tubes lacking *O*-glycosylation exhibit altered F-actin organization

**Fig. 2.**
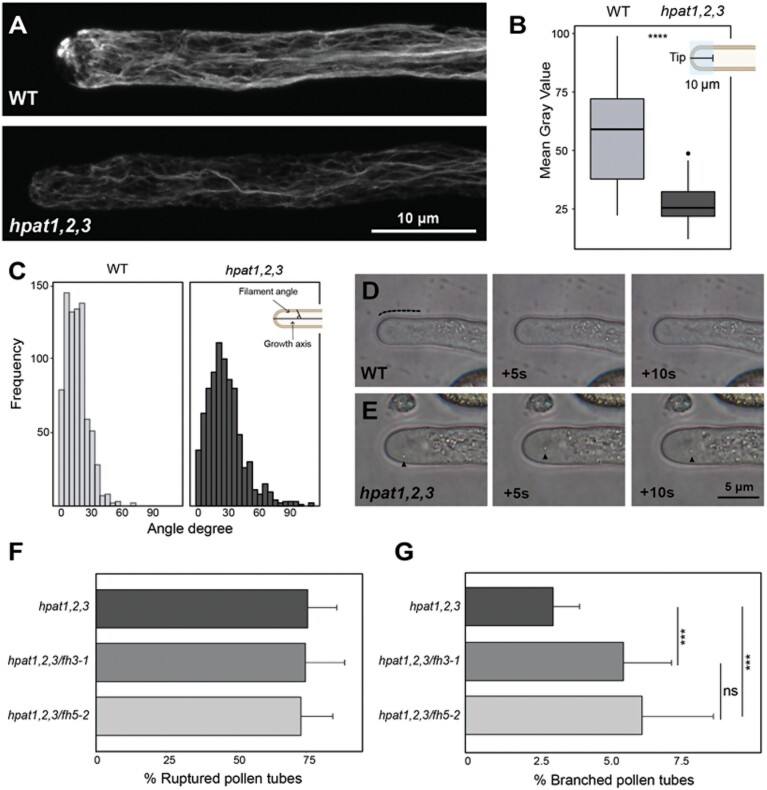
Pollen tubes lacking *O*-arabinosylation display F-actin cytoskeleton disorganization. (A) Representative images of *in vitro* germinated pollen tubes stained with phalloidin-iFluor 488. Top, wild-type; bottom, *hpat1,2,3*. (B) Quantification of fluorescence intensity in the apical–subapical region (10 μm from tip dome toward the shank, blue shaded square in top right schematic diagram). *n*=20 for each genotype; ****P<0.0005: statistically significant difference (Student’s *t*-test). (C) Actin filament angle distribution in wild-type (left) and mutant (right) pollen tubes. Angles were measured with respect to the growth axis (top right schematic diagram); over 700 filaments from 20 tubes were measured for each genotype. (D) Wild-type pollen tubes grown *in vitro* (*n*=14). The apical ‘clear zone’ is indicated with a dashed line. (E) Invasion of the apical clear zone in *hpat1,2,3* pollen tubes grown *in vitro*. Micrographs represent time series from the same pollen tube; arrowhead indicates invasive particle (*n*=17). (F–G) Phenotyping of triple *hpat1,2,3/fh3-1* and *hpat1,2,3/fh5-2* mutants. Compared with *hpat1,2,3* pollen tubes, *hpat1,2,3/fh3-1* and *hpat1,2,3/fh5-2* mutants showed increased pollen tube branching (F) but not increased pollen tube rupture (G). Mean and SD of pollen branching/rupture measured after 3 h of germination *in vitro*, >5 biological replicates per genotype (*n*>2500); ***P_adjusted_<0.005: statistically significant difference (Student’s *t*-test); ns, no statistically significant difference.

Evidence for the extent of cytoskeleton, plasma membrane, and cell wall interconnection is often provided by reciprocal defects in cytoskeleton organization or cell wall structure when one is disrupted (Chebli *et al.*, 2021). Our group recently showed that *hpat* mutant pollen tubes exhibit an altered distribution of cell wall polymers (Beuder *et al.*, 2020). Based on our findings on the localization of AtFH5 in the *hpat1,2,3* background, we predicted that mutant pollen tubes will exhibit actin disorganization, potentially as a result of AtFH5 mislocalization (Fig. 1). We analysed the organization of the actin cytoskeleton in pollen tubes grown *in vitro* by fluorescent labeling of actin filaments with phalloidin (Fig. 2). Compared with wild-type tubes, mutant pollen tubes displayed a significant decrease in fluorescence intensity (Fig. 2A, B). In addition, *hpat1,2,3* pollen tubes showed distinct organization of F-actin compared with wild-type pollen tubes (Fig. 2A). To quantitatively assess the extent of actin disorganization in mutant pollen tubes, we measured the angles formed by individual filaments in semi-projections (two to three adjacent *z*-slices) relative to the axis of growth throughout the pollen tube width. In wild-type pollen tubes, most filaments formed acute angles (≤30°), oriented almost parallel to the growth axis. Actin filaments in *hpat1,2,3* pollen tubes, on the other hand, formed angles in a wider degree range, including 90° angles, which were absent in the wild-type (Fig. 2C). In wild-type pollen tubes, large organelles (lipid droplets, amyloplasts, Golgi) remain restricted to the shank, while the tip is enriched in small endo/exocytic vesicles (Fig. 2D), forming a region known as the ‘clear zone’ (Cheung and Wu, 2007). Proper organization of the actin cytoskeleton in pollen tubes is important to maintain the cytoplasmic zonation; disruption of actin disorganization often leads to invasion of the clear zone (Li *et al.*, 2018). In *hpat1,2,3* pollen tubes, we observed occasional invasion of the clear zone by large particles displaying erratic movement over time (~23% of tubes, *n*=17, Fig. 2E). Clear zone invasion was not observed in any of the analysed wild-type pollen tubes (*n*=14).

The F-actin organization defects observed in *hpat1,2,3* pollen tubes are consistent with potential ectopic AtFH5 activity due to mislocalization and/or potential interference with AtFH3 activity. Alternatively, although not mutually exclusive, F-actin disorganization could be a consequence of the altered cell wall organization in mutant pollen tubes (Beuder *et al.*, 2020), ultimately altering the linkage between cell wall–actin cytoskeleton and disrupting cell polarity. To better understand the potential genetic interactions between pollen class I formins, particularly AtFH5, and HPATs, we generated quadruple mutants *hpat1,2,3/fh3-1* and *hpat1,2,3/fh5-2* and evaluated the effects on pollen tube morphology compared with the *hpat1,2,3* mutant. Interestingly, phenotypic analyses of the quadruple mutants showed a significant increase in pollen tube branching compared with the *hpat1,2,3* background while the percentage of pollen tube bursting in the triple mutants remained unchanged (Fig. 2F, G), suggesting that pollen tube branching is potentially a consequence of altered F-actin dynamics rather than compromised cell wall integrity.

As determined earlier, the mNG fusions of AtFH3 and ECD modified versions did not exhibit an altered plasma membrane localization in the *hpat1,2,3* mutant background (Fig. 1D–I). Considering that the ECD of AtFH3 contains AGP-like motifs, we investigated whether interfering with AGP glycosylation and potentially the post-translational modification of the ECD of AtFH3 could also have an effect in F-actin organization. The β-Yariv reagent binds selectively to the β-1,3-galactan main chains of AGPs, precipitating them (Kitazawa *et al.*, 2013). β-Yariv is not only used to precipitate AGPs *in vitro*, but also to perturb AGP function *in vivo* (Přerovská *et al.*, 2021). Thus, we incubated *in vitro-*grown pollen tubes with 30 μM β-Yariv and stained the actin cytoskeleton with fluorescent phalloidin. Altered pollen tube morphology and growth arrest was observed after treatment, while F-actin organization, particularly in the apical and subapical region, was altered (see Supplementary Fig. S3A, B). Furthermore, to simultaneously disturb *O*-glycosylation of AGP and EXT, and potentially the ECDs of AtFH3/5, we evaluated the effect on actin organization of treatment with 3,4-dehydro-DL-proline (3,4-DHP). 3,4-DHP is a selective inhibitor of prolyl hydroxylases, thus effectively disrupting *O*-glycosylation of HRGPs (Zhang *et al.*, 2014). The three tested concentrations of 3,4-DHP tested (10, 20 and 30 μM) induced changes in pollen tube morphology (i.e. branching and bulging), as well as an altered distribution of actin filaments (Supplementary Fig. S3C–F). Taken together, these results suggest that F-actin organization in elongating pollen tubes is sensitive to perturbations in HRGP *O*-glycosylation pathways, possibly affecting the function of pollen class I formins.

### AtFH5’s apical plasma membrane localization is maintained by endocytosis

**Fig. 3.**
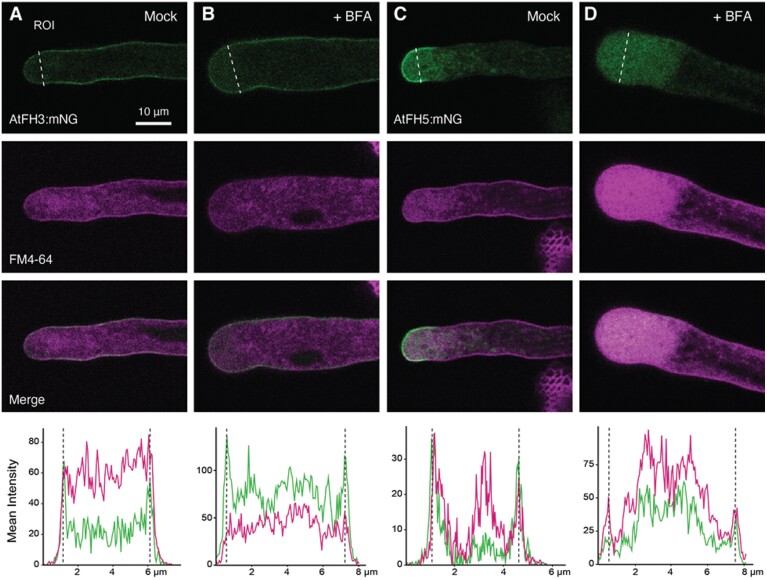
AtFH5 is subapically internalized by endocytosis. Representative images of pollen tubes expressing AtFH3:mNG (A, B) or AtFH5:mNG (C, D) treated with brefeldin A (BFA). Pollen tubes were grown *in vitro* and stained with the lipophilic dye FM4-64 (12 µM). Pollen tubes were then incubated with BFA (25 µM in liquid germination medium) (B, D) or a mock treatment (methanol in liquid germination medium) (A, C) for 60 min (*n*>20 per construct and treatment). The plots at the bottom show mean intensity quantification for mNG signal (green) or FMF-64 (magenta) in an ROI traced with white dashed line across the pollen tube width in the subapical region of the pollen tube for each treatment; dashed lines represent the edges of the pollen tube.

Our results indicate that the apically restricted localization of AtFH5 in elongating pollen tubes is dependent on the ECD and its potential post-translational modification by HPATs (Fig. 1). However, other mechanisms such as endocytosis are known to limit the distribution of other secreted, tip-localized proteins in pollen tubes (Röckel *et al.*, 2008; Grebnev *et al.*, 2017). To investigate whether endocytosis is involved in the observed patterning of AtFH3 and AtFH5, we evaluated the effects of brefeldin A (BFA) treatment on their localization in *in vitro-*grown pollen tubes. The fungal metabolite BFA is known to disrupt plant cell membrane trafficking by blocking exocytosis while allowing endocytosis to occur (Baluška *et al.*, 2002). Pollen tube growth is arrested when treated with BFA, inducing the accumulation of FM4-64 positive membrane aggregates in the subapical region (BFA-induced aggregates, BIAs) (Parton *et al.*, 2003). Reports suggest that treatment also enhances endocytosis in pollen tubes (Wang *et al.*, 2005). Therefore, we hypothesized that if endocytosis played a role in AtFH5 localization, BFA treatment would cause intracellular accumulation of AtFH5, co-localizing with BIAs. Pollen tubes expressing either AtFH3:mNG or AtFH5:mNG were stained with FM4-64 and then treated with BFA for 60 min. After treatment, we observed that AtFH5:mNG was completely depleted from the plasma membrane, accumulating in the subapical region and co-localizing with FM4-64 stain (Fig. 3C, D). The localization of AtFH3, on the other hand, remained unchanged compared with the mock treatment (Fig. 3A, B). These results suggest endocytic internalization of AtFH5 in the pollen tube’s shank participates in restricting its accumulation beyond the apical plasma membrane.

### The extracellular domains of class I formins bear distinct types of *O*-glycans

**Fig. 4.**
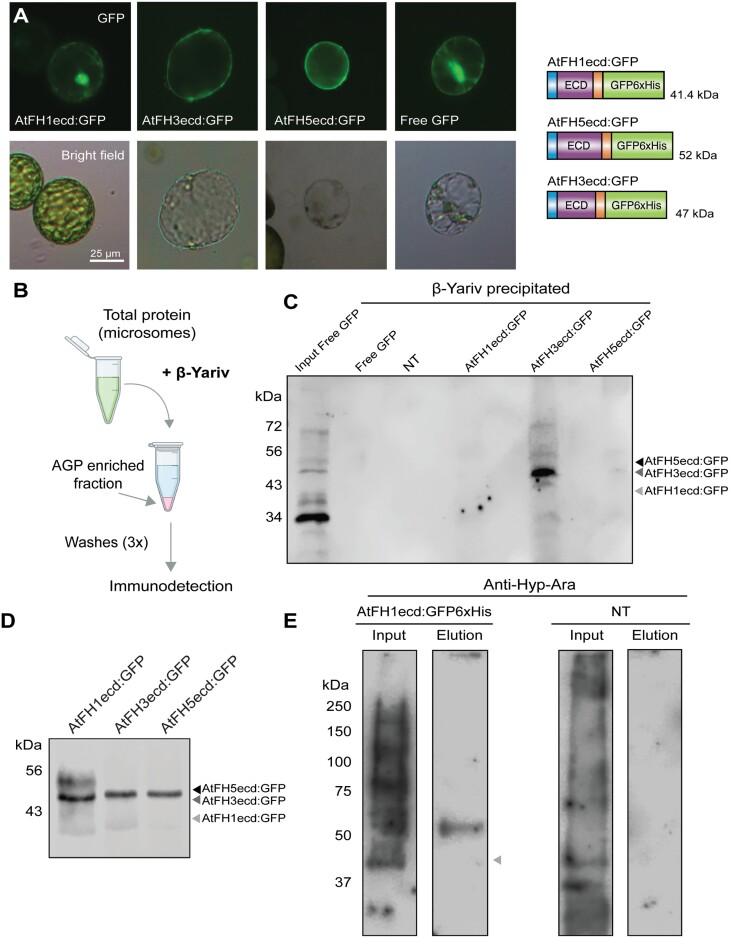
The HRGP-like motifs in the ECDs of class I formins are *O*-glycosylated. (A) GFP-tagged ECDs of AtFH1 (AtFH1ecd:GFP), AtFH3 (AtFH3ecd:GFP), and AtFH5 (AtFH5ecd:GFP) are localized to the plasma membrane of protoplasts isolated from transiently transformed tobacco leaves. On the right, protein schematic diagram of GFP tagged ECDs and their predicted sizes. (B) Schematic representation of precipitation of AGP and AGP-like proteins from total protein fractions with the β-Yariv reagent. (C) Immunodetection of β-Yariv precipitated proteins with anti-GFP antibody. Microsomes used as input for all samples was isolated from agroinfiltrated tobacco leaves. NT, non-transformed control. (D) Immunodetection with anti-GFP antibody of GFP-tagged ECDs from total microsomal fractions derived from Arabidopsis stable lines. (E) Left, immunodetection of Hyp-arabinosylation of total microsomal fractions derived from plants expressing AtFH1ecd:GFP6×His (input, 20 µg) or His-purified FH1ecd:GFP6×His (2.25% of total eluate). A band corresponding to the band observed for FH1ecd:GFP6×His in (D) was detected when probing with anti-Hyp-Ara antibody. Right, as a negative control, microsomes derived from non-transformed plants (NT) were probed with anti-Hyp-Ara. No visible bands are detected in the eluate after His-purification of NT samples. Predicted sizes of the protein backbone are marked with arrowheads.

*O*-glycosylation of the ECD of class I formins has long been speculated (Banno and Chua, 2000; Borassi *et al.*, 2016); however, direct evidence is still lacking. To address this question, we generated genetically tagged versions of the ECD of pollen formins AtFH3 (AtFH3ecd:GFP6×His) and AtFH5 (AtFH5ecd:GFP6×His), and also included the vegetative formin, AtFH1 (AtFH1ecd:GFP6×His) (Fig. 4B). These constructs contained their respective signal peptides and transmembrane domains, and as expected, localized to the plasma membrane when expressed transiently in tobacco leaves (Fig. 4A). Immunodetection of the tagged ECDs in the microsomal fraction isolated from stable Arabidopsis lines showed higher sizes than predicted based on the amino acid sequences (Fig. 4D), potentially indicating the presence of post-translational modifications on the protein backbones.

According to the hydroxyproline contiguity hypothesis (Shpak *et al.*, 2001) and based on the amino acid sequences of the ECDs of the selected formins (Fig. 2B), we hypothesized that the ECD of AtFH3 will bear AGP-like glycans, while the ECDs of AtFH1 and AtFH5 will be primarily *O*-arabinosylated. To address their glycosylation status, we followed two strategies: first, we investigated the presence of AG glycans in the ECDs by utilizing the synthetic reagent β-Yariv (Fig. 4B). Microsomal fractions derived from transiently transformed tobacco leaves expressing the GFP-tagged ECDs of AtFH1, AtFH3, or AtFH5 were incubated with β-Yariv, and the resulting AGP-enriched fractions were analysed by immunodetection. Our results showed that among all the analysed ECDs, AtFH3ecd:GFP6×His was greatly enriched in the fraction precipitated by β-Yariv. Free GFP did not display reactivity towards the β-Yariv reagent, demonstrating the specificity of this assay (Fig. 4B, C).

The second approach consisted of protein purification by metal affinity chromatography followed by immunodetection with anti-glycan antibodies. While we attempted to purify all three ECDs from Arabidopsis stable lines (Fig. 4D), an acceptable level of purity to allow glycoprofiling was only achieved for AtFH1ecd:GFP6×His (see Supplementary Fig. S4C). After protein purification, AtFH1ecd:GFP6×His was probed with two anti-glycan antibodies: anti-Hyp-Ara (JIM19) and anti-Hyp-AG (JIM13) (Knox *et al*., 1991, 1995; Yates and Knox, 1994). Hyp-*O*-arabinosylation was detected in the input of microsomes derived from both plants expressing AtFH1ecd:GFP6×His and non-transformed plants (Input, Fig. 4E); however, after His-purification, glycosylation was only detected in the eluate of AtH1ecd:GFP6×His (Elution, Fig. 4E). Supporting our hypothesis, the observed band in the AtFH1ecd:GFP6×His corresponds to the size observed when probing with anti-GFP (Fig. 4D). Consistent with the β-Yariv precipitation assay, purified AtFH1ecd:GFP6×His did not show reactivity to anti-Hyp-AG antibodies (Supplementary Fig. S4C, D), supporting our hypothesis.

HRGP-like motifs are present in the ECDs across most members of the class I formin family (see Supplementary Fig. S5, Borassi *et al.*, 2016). In addition to AtFH1 and AtFH5, AtFH9 and AtFH10 possess EXT-like motifs; AtFH11, like AtFH3, contains exclusively AGP-like motifs. The remaining members of the family (AtFH2, AtFH4, AtFH6, and AtFH8) possess short AGP-like clustered dipeptides (two to three repeats) and Ser-Pro-Pro repeats that, like the Ser-Pro_(3–5)_ EXT motifs, can be modified by the addition of arabinosides (Shpak *et al.*, 2001; Estévez *et al.*, 2006). Taken together, our results provide the first direct biochemical evidence suggesting that the chimeric HRGP motifs present in the ECDs of members of the class I formin family are *O*-glycosylated and that these post-translational modifications might be widespread across the clade.

### The extracellular domains of pollen class I formins display distinct lateral plasma membrane mobility

Integral membrane (or plasma membrane associated) proteins facing the cell wall display restricted lateral mobility (Martinière *et al.*, 2012). AtFH1, a class I formin expressed in vegetative tissues, is immobilized in the plasma membrane by its interaction with the cell wall (Martinière *et al.*, 2011). Based on these observations, class I formins have been regarded as candidates to mediate physical membrane anchoring to the wall or Hechtian adhesion (Pont-Lezica *et al.*, 1993; Lamport *et al.*, 2018). In addition, AGPs were reported to co-localize with membranous thread-like structures (Hechtian strands) upon plasmolysis, thus having a potential role in Hechtian adhesion (Sardar *et al.*, 2006). Similarly, canonical EXTs have been shown to serve as a scaffold for pectin supramolecular assembly and to covalently crosslink with pectin polysaccharides (Cannon *et al.*, 2008), also having the potential to establish interactions with the wall through a distinct mechanism. Given that our biochemical studies provided evidence for the presence of AGP-like glycans in the ECD of AtFH3 and putative EXT-like glycans in the ECD of AtFH5 (Fig. 4), we investigated whether Hechtian adhesion in pollen tubes was reduced in the loss of function alleles *fh3-1* and *fh5-2*. Plasmolysed wild-type, *fh3-1* and *fh5-2* pollen tubes were stained with the lipophilic dye FM4-64 and imaged by confocal microscopy. Although FM4-64 positive structures were observed to localize in the apoplastic space upon plasmolysis in all three genotypes, a quantitative assessment of the extent of adhesion among genotypes was limited due to the size of the Hechtian strands, which were below the resolution achieved by confocal microscopy (see Supplementary Fig. S6A). Neither AtFH3 nor AtFH5 have been reported to localize to Hechtian strands in pollen tubes, and therefore we induced plasmolysis in our complemented lines (*fh3-1 C* and *fh5-2 C*) and stained them with FM4-64. We found that AtFH3:mNG colocalized with FM4-64 positive membranous extensions that remained in contact with the wall upon plasmolysis. AtFH5:mNG signal was also detected in the apoplastic space, but only partial colocalization was observed (Supplementary Fig. S6B). These results indicate that the ECDs of AtFH3 and AtFH5 establish distinct types of interactions with the wall, with AtFH3 and AG glycans in their ECD possibly involved in Hechtian adhesion in elongating pollen tubes.

Having determined that both AtFH3 and AtFH5 establish interaction with the wall (see Supplementary Fig. S6B) and based on our localization studies, particularly the expansion of AtFH5 plasma membrane localization in the *hpat1,2,3* mutant background (Fig. 2J, N), we asked whether the presence of distinct types of glycans in their ECDs (Fig. 4) has implications in their lateral mobility.

**Fig. 5.**
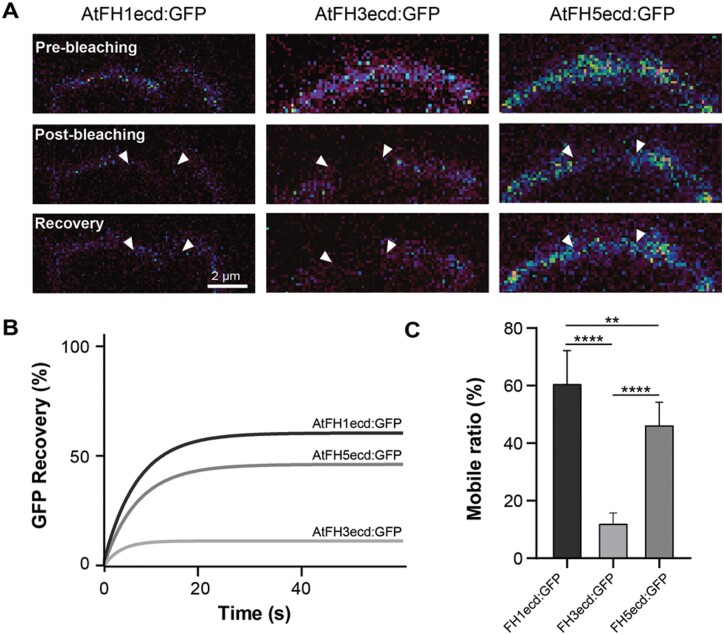
The ECDs of class I formins exhibit different degrees of lateral mobility. (A) Representative images of fluorescence recovery after photobleaching (FRAP) assays in Arabidopsis epidermal cells expressing AtFH1ecd:GFP, AtFH3ecd:GFP, or AtFH5ecd:GFP. These constructs correspond to those depicted in Fig. 4B. White arrowheads represent the boundaries of the photobleached area and recovery images represent the last time point captured after photobleaching (60 s). (B) FRAP curves revealed very low mobility for AtFH3ecd:GFP compared with AtFH1ecd:GFP and AtFH5ecd:GFP. (C) Quantification of the mobile fraction of AtFH1ecd:GFP (*n*=8), AtFH3ecd:GFP (*n*= 9). and AtFH5ecd:GFP (*n*=11). **P_adjusted_<0.005, ****P_adjusted_<0.0005: statistically significant (Student’s *t*-test).

First, we investigated the lateral mobility of the ECDs of AtFH1, AtFH3, and AtFH5, eliminating the influence of their respective intracellular domains. We performed FRAP assays in epidermal cells of Arabidopsis leaves expressing the ECD GFP-tagged versions (Fig. 4A, B). As expected for cell wall-interacting plasma membrane proteins and consistent with Martinière *et al.* (2011, 2012), all three constructs exhibited limited lateral diffusion; however, significant differences in the degree of recovery were observed among ECDs: AtFH3 exhibited almost no recovery of fluorescence post-photobleaching (mobile fraction 12 ± 3.6%, *n*=9), while AtFH5 and AtFH1 displayed comparatively higher recovery (mobile fraction 46.5 ± 8.1%, *n*=11 and 60.9 ± 11.6%, *n*=8, respectively) (Fig. 5). AtFH1 and AtFH5, both containing EXT-like motifs in their ECDs, displayed similar recovery curves and lateral mobility. In contrast, AtFH3, which contains AGP-like glycomodules was highly restricted, suggesting that the different glycans attached to these proteins (Fig. 4C, E) greatly influence their mobility.

**Fig. 6.**
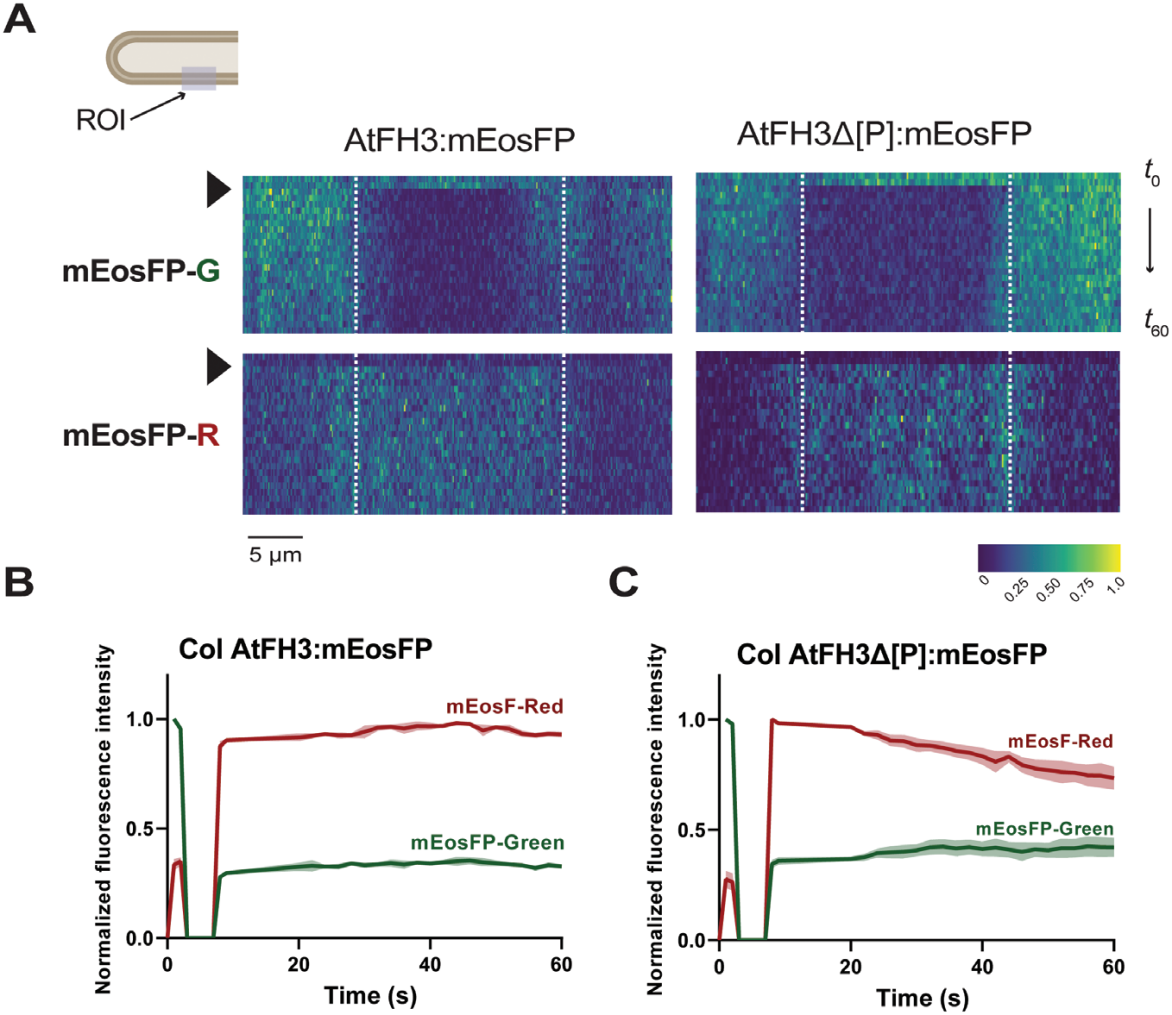
Interaction of AtFH3 with the actin cytoskeleton limits its lateral diffusion. (A) Lateral diffusion dynamics of AtFH3:mEosFP (left) or AtFH3Δ[P]:mEosFP (right) after photoconversion in the wild-type background. Kymographs represent the normalized fluorescence intensity in the photoconverted region (ROI indicated in pollen tube schematic diagram corresponds to the region delineated with dashed white lines within kymographs) and surrounding area for the green form of mEosFP (mEosFP-G, top panels) or photoconverted red form of mEosFP (mEosFP-R, bottom panels) over time (*t*). Black arrowhead indicates the time of photoconversion. The color scale indicates the normalized fluorescence intensity from 0 to the highest intensity value possible, 1. (B) Quantification of AtFH3 mean normalized fluorescence intensity (colored lines) of mEosFP-G or mEosFP-R in the ROI, pre- and post-photoconversion and standard error (shading), *n*=8. (C) Quantification of AtFH3Δ[P]:mEosFP-G or AtFH3Δ[P]:mEosFP-R pre- and post-photoconversion; *n*=7.

Next, we investigated the effect of the F-actin cytoskeleton in protein anchoring *in situ*. Considering that AtFH3 interacts with a much more stable subarray of actin bundles in the pollen tube’s shank, while AtFH5 participates in the nucleation of highly dynamic, finer actin filaments in the apical and subapical region (Cheung *et al.*, 2010; Qu *et al.*, 2015), we sought to determine whether this interaction has an effect on the protein lateral diffusion or if, as reported for AtFH1 in epidermal cells (Martinière *et al.*, 2012), the ECD is primarily responsible of protein anchoring (Fig. 5). Due to the high photostability of the mNG protein observed when attempting FRAP experiments, we generated translational fusions of the full length AtFH3/5 and the Pro-rich region deletions (Δ[P], Fig. 1A, B) with the photoconvertible protein mEosFP (Mathur *et al.*, 2010). Upon exposure to blue light, mEosFP irreversibly switches its emission spectrum from green to red. Lateral diffusion of the green mEosFP (mEosFP-G) is tracked over time akin to FRAP experiments but without potential cell photodamage (Wozny *et al.*, 2012). We predicted that if protein anchorage is dependent on the ECD, Δ[P]:mEosFP fusions might display an increase in their lateral diffusion compared with their full length counterparts. Kymographic analyses revealed no difference in lateral diffusion pattern between AtFH3:mEosFP and AtFH3Δ[P]:mEosFP (Fig. 6), suggesting that both the ECD and the intracellular domains participate in anchoring AtFH3 to the plasma membrane. In the case of AtFH5, we observed an overall higher lateral mobility compared with AtFH3; however, the patterning observed in the kymographic analysis and high mEosFP-G recovery (see Supplementary Fig. S7) might be partially due to rapid pollen tube elongation and continuous secretion of non-photoconverted protein. Thus, the contribution of the actin cytoskeleton in AtFH5 remains to be determined. The observed influence of the actin cytoskeleton on protein mobility in AtFH3 is consistent with its involvement in nucleation/bundling of actin filaments in the pollen tube shank (Thomas, 2012; Qu *et al.*, 2015).

## Discussion

Here, we provide functional insights on the ECD of class I formins and their role as molecular linkers mediating the crosstalk between the cell wall, plasma membrane, and actin cytoskeleton. The study of AtFH3 and AtFH5 in elongating pollen tubes offered a unique system to evaluate the functional significance of the ECD of class I formins, as both AtFH3 and AtFH5 are expressed in the same cell structure and yet display a unique spatial patterning (Fig. 1D, J). While differences in their intracellular actin nucleation activities, differential affinity to profilin, or other unknown interactors might modulate their respective patterning (Cheung *et al.*, 2010; Thomas, 2012; Lan *et al.*, 2018; Liu *et al.*, 2021), our results suggest a pivotal role for the ECD in their localization. This is evidenced by the failure of the ΔECD:mNG versions to localize to the plasma membrane and/or the inability to rescue the germination defect or actin organization in their respective transcriptional null backgrounds (Fig. 1E, K; Supplementary Figs S1, S2). Interestingly, reports on other members of the family indicate that the ECD is required for their localization in different cellular structures: the ECD of AtFH8 is necessary for translocation from the nucleus to the newly formed cell wall after cell division (Xue *et al.*, 2011), while the ECD of AtFH2 is necessary for plasmodesmata localization in epidermal cells (Diao *et al.*, 2018), suggesting that the ECD plays a role in protein localization not only in pollen-expressed formins but might be required for proper plasma membrane localization across the class I family. Furthermore, our study provides evidence for the presence of post-translational modifications of the HRGP-like glycomotifs present in the ECDs of two members of the family (Fig. 4), following the predictions of the hydroxyproline contiguity hypothesis (Shpak *et al.*, 2001) and setting the precedent of *O*-glycosylation for other members of the class I formin family and possibly other HRGP chimeras (Leucin-Rich Repeat Extensins, Proline-rich Extensin-like Receptor Kinases, etc.). Naturally, the next question relates to the significance of these post-translational modifications in the protein’s function. Our data indicate that the ECDs containing EXT-like motifs (AtFH1, AtFH5) exhibit increased lateral mobility relative to the ECD of AtFH3, which contains AGP-like motifs (Fig. 5). Although the underlying mechanism requires further investigation, these results raise intriguing scenarios. HRGPs exhibit virtually all the properties that define Intrinsically Disordered Proteins (IDPs): high content of Pro residues in their sequences, repetitive motifs, and the presence of PTMs in such motifs (Johnson *et al.*, 2017); these features confer flexibility to the protein’s conformation and structural plasticity, permitting transient molecular interactions (Uversky, 2019). Increasing evidence indicates that IDPs and disordered regions have important roles in cellular signaling (Sun *et al.*, 2012; Hsiao *et al.*, 2020). In particular, classical EXTs and AGPs are believed to play antagonistic roles in cell wall polysaccharide remodeling (Lamport *et al.*, 2011). EXTs form supramolecular networks that serve as a scaffold for the assembly and crosslinking of pectin in the primary cell wall (Showalter and Basu, 2016), whereas AGPs putatively act as pectin plasticizers by regulating availability of Ca^2+^ in the periplasm (Lamport *et al.*, 2018; Lopez-Hernandez *et al.*, 2020; Silva *et al.*, 2020). Therefore, if the ECD of class I formins shares dynamic structural features of IDPs and known biochemical properties of EXTs and/or AGPs, they might interact with cell wall polysaccharides or other extracellular (glyco)proteins, potentially establishing a cell wall sensing module. Supporting this hypothesis, we provide evidence that both AtFH3 and AtFH5 interact with the wall and that, upon plasmolysis, both exhibit apoplastic localization, with AtFH3 primarily colocalizing with plasma membrane extensions that remain anchored to the wall (Supplementary Fig. S6B), suggesting a potential role for Hechtian adhesion, a mechanism proposed to act as an important mechanotransduction mechanism during tip growth (Lamport *et al.*, 2018).

Finally, polarized growth in pollen tubes requires coordination between cell wall assembly and F-actin dynamics. Decoupling of these processes leads to disruption of growth, as observed in *hpat* mutant pollen tubes (MacAlister *et al.*, 2016, Fig. 2). Although *hpat1,2,3* pollen tubes exhibit compromised cell wall integrity most likely due to the lack of arabinosylation of their canonical targets, EXTs (Beuder *et al.*, 2020), we provide genetic and biochemical evidence of novel chimeric targets that establish a linkage between cell wall, plasma membrane, and actin cytoskeleton. Our data indicate that AtFH5 is maintained at the apical membrane by endocytosis (Fig. 3) and the lack of Hyp-*O*-arabinosylation alters its patterning (Fig. 1). Whether the EXT-like motifs in AtFH5 are directly modified remains to be determined; however, we were able to detect Hyp-*O*-arabinosylation in AtFH1, another class I formin with EXT-like motifs (Fig. 4E), suggesting that these motifs might be modified by HPATs. In the case of the ECD of AtFH3, we were able to show reactivity to the β-Yariv reagent, indicating the presence of arabinogalactan glycans (Fig. 4B, C). While the ECD alone restricts protein mobility in epidermal cells (Fig. 5), we also found that in highly polarized and fast-growing cells like pollen tubes, the actin cytoskeleton also plays an important role in immobilizing AtFH3, to the plasma membrane, possibly through Hechtian adhesion (Fig. 6; Supplementary Fig. S6B). Although both pollen formins had been demonstrated to have *in vitro* actin nucleation activity (Ingouff *et al.*, 2005; Ye *et al.*, 2009), a recent genetic study showed that AtFH5’s activity is enhanced by pollen-expressed reproductive profilins 4 and 5 (PRF4 and PRF5) during the formation of a collar-like structure in germinating pollen grains (Liu *et al.*, 2021). *In vitro* studies show that profilin has an enhancing effect on formin activity, forming pools for fast nucleation and polymerization of actin filaments (Romero *et al.*, 2004). While the effect of PRF4/5 on AtFH3’s activity remains to be investigated, these observations open a scenario in which AtFH5 participates in rapid nucleation/polymerization of cortical actin of highly dynamic apical and subapical actin arrays in pollen tubes and its ECD accounts for the protein’s anchoring, while AtFH3 is anchored to the shank of the pollen tube by its ECD and association with more stable axial actin filaments.

## Supplementary data

The following supplementary data are available at *JXB* online.

Fig. S1. Loss of function alleles of AtFH3 and AtFH5 exhibit a reduction in germination but not growth rate.

Fig. S2. The ECD of AtFH3 and AtFH5 is necessary to restore apical actin levels in *fh3-1* and *fh5-2* pollen tubes.

Fig. S3. Chemical-induced disruption of *O*-glycosylation alters F-actin organization in pollen tubes.

Fig. S4. Validation of anti-Ara antibody used for glycoprofiling, and absence of AG-glycans in purified AtFH1ecd.

Fig. S5. HRGP-like motifs are present in the ECDs of members of the class I formin family.

Fig. S6. AtFH3 and AtFH5 exhibit partial co-localization with membranous extensions upon plasmolysis in pollen tubes.

Fig. S7. AtFH5:mEosFP exhibits a high degree of lateral mobility.

Table S1. Primers used in this study.

Table S2. Linear mixed effects model parameters and model fit output.

erac131_suppl_Supplementary_Figures_S1-S7_Tables_S1-S2

## Data Availability

The data supporting the findings of this study are available from the corresponding author, CAM, upon request.

## Abbreviations

FH: formin homology
ECD: extracellular domain
HRGP: hydroxyproline-rich cell wall glycoprotein
