# Supplementary material for: O-glycosylation of the extracellular domain of pollen class I formins modulates their plasma membrane mobility
Source: J Exp Bot. 2022 Apr 6;73(12):3929–45. doi: 10.1093/jxb/erac131 (PMC9232206; doi:10.1093/jxb/erac131)
Supplement: erac131_suppl_Supplementary_Figures_S1-S7_Tables_S1-S2 [file erac131_suppl_supplementary_figures_s1-s7_tables_s1-s2.pdf]

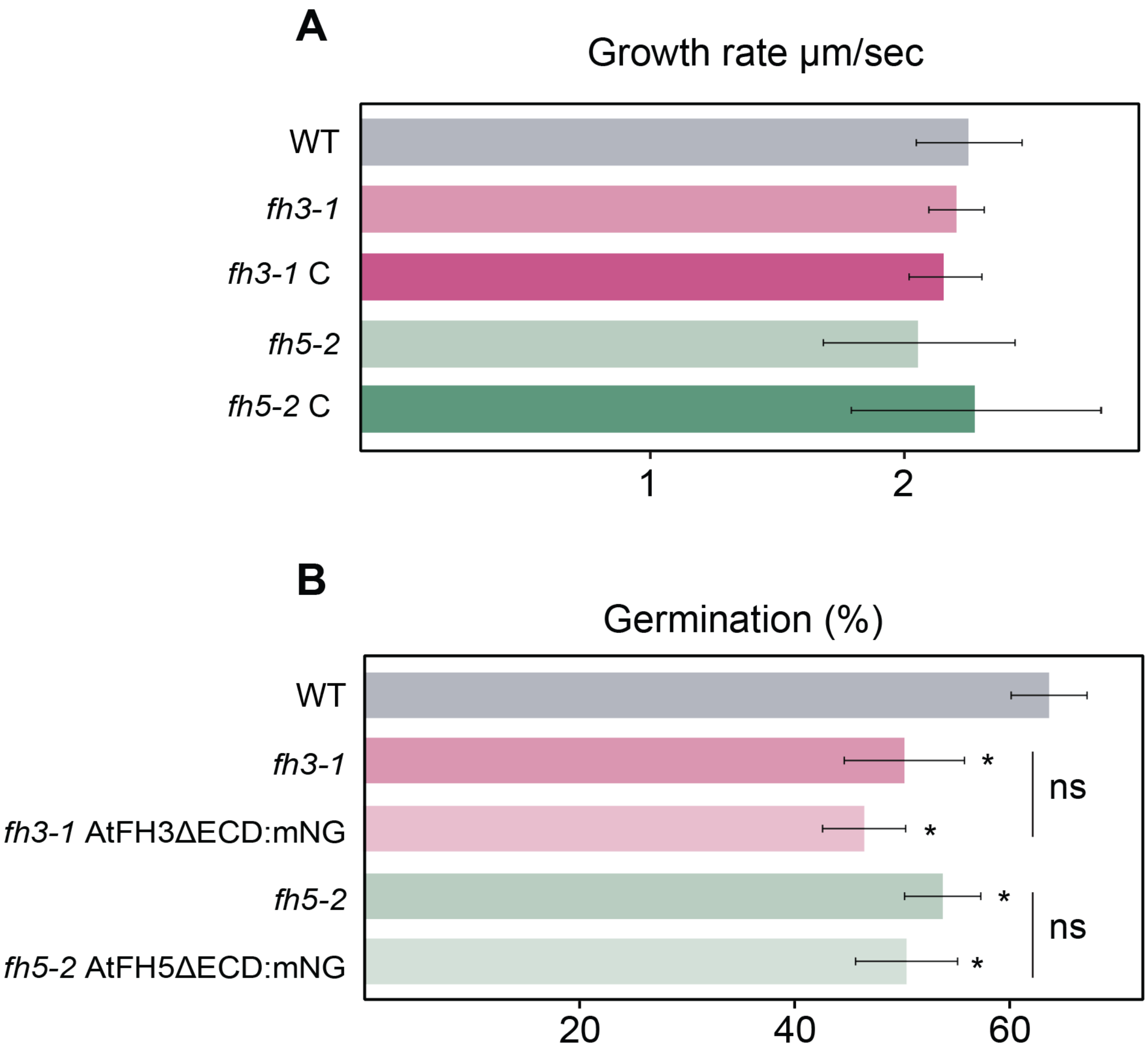

Figure S1. A) Quantification of pollen tube growth rate in *fh3-1*, *fh5-2* and complemented lines with AtFH3 or AtFH5 translational fusions with mNG, no statistically significant differences in growth rate compared to wild-type pollen tubes were detected. Growth rate was measured over a period of 15 min. **B)** Introgression of AtFH3ΔECD:mNG or AtFH5ΔECD:mNG into *fh3-1* or *fh5-2,* respectively, does not rescue their germination defect. Pollen germination *in vitro* was measured after 3 h. Three biological replicates per genotype, '*' statistically significant difference (Student's T-test adjusted p-value < 0.05, n > 1000). 'ns' indicates no statistically significant difference.

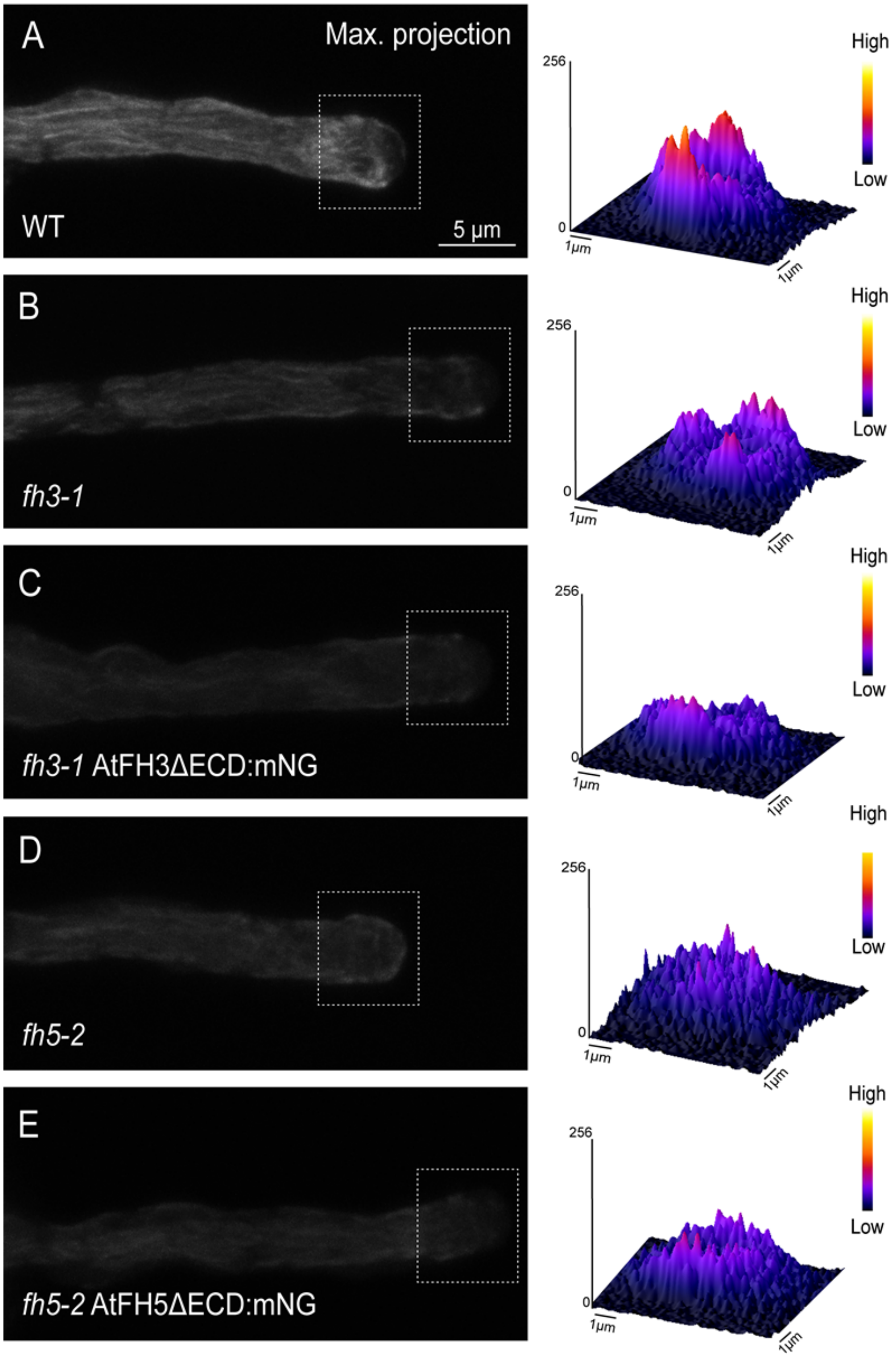

Figure S2. AtFH3 **(A)** and AtFH5 **(B)** loss of function alleles and ECD deleted versions **(D, E)** exhibit reduced pollen tube actin labeling. *In vitro* grown pollen tubes (N≥10 per genotype) were labeled with Phalloidin-iFluor 594. On the left, surface 3D plots of the distribution of fluorescence intensity values within the apical region (white dashed box). Color scale represents pixel intensity gray value (Low=0, High=256).

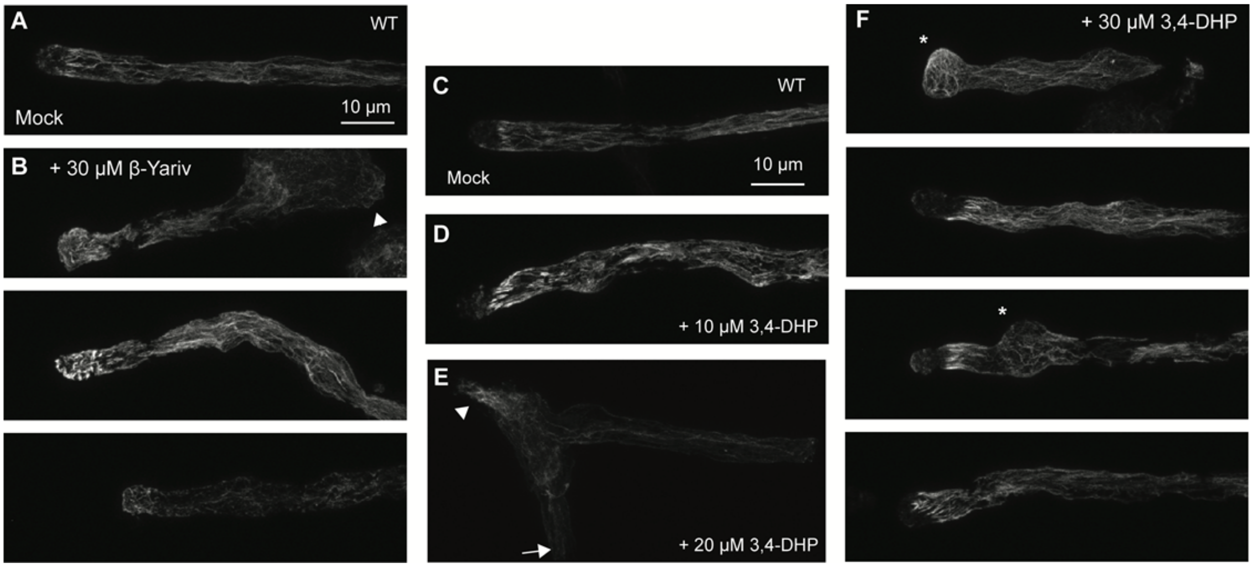

Figure S3. Disruption of *O*-glycosylation alters F-actin organization in pollen tubes. *In vitro* grown wild-type pollen tubes (WT) were incubated with a mock treatment **(A,C),** 30 µM β-Yariv **(B)**, 10 µM 3,4 DHP **(D),** 20 µM 3,4-DHP (**E**) or 30 µM 3,4-DHP **(F)** and then the actin cytoskeleton was stained with Phalloidin-iFluor 488 (*n*=10 per treatment). Secondary tips are indicated with white arrowheads, '*' indicates bulging, and in D, white arrow indicates primary tip.

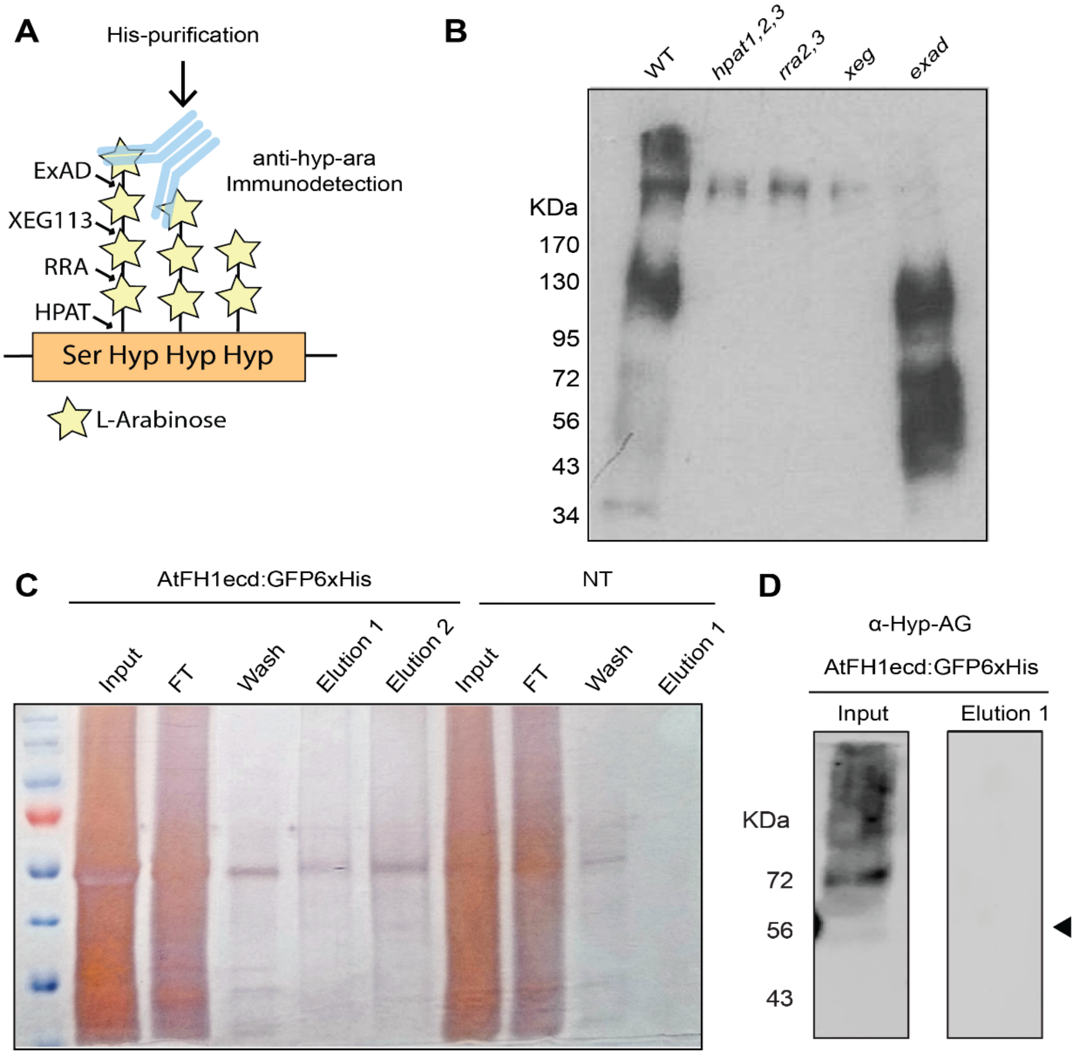

Figure S4. **A)** Strategy for glycoprofilling purified ECD domains. **B)** Hyp-*O*-ara antibody validation. Total protein extracts from Columbia wild type, *hpat1,2,3*, *rra2,3*, *xeg* or *exad* seedlings were probed with the anti-hyp-O-ara antibody JIM19. After HPATs add the first arabinose sugar, the linear chain is serially extended by the glycosyltransferases indicated in A. Loss of signal is observed in protein extracts derived from *hpat1,2,3, rra2,3* or *xeg* seedlings, suggesting that the epitope of this antibody is a linear arabinose chain of at least three sugars. **C)** Silver staining of fractions collected during His purification of AtFH1ecd:GFP6xHis or a non-transformed (NT) negative control. **D)** Input and Elution 1 from AtFH1ecd:GFP6xHis was probed with an anti-hyp-AG antibody (JIM13).

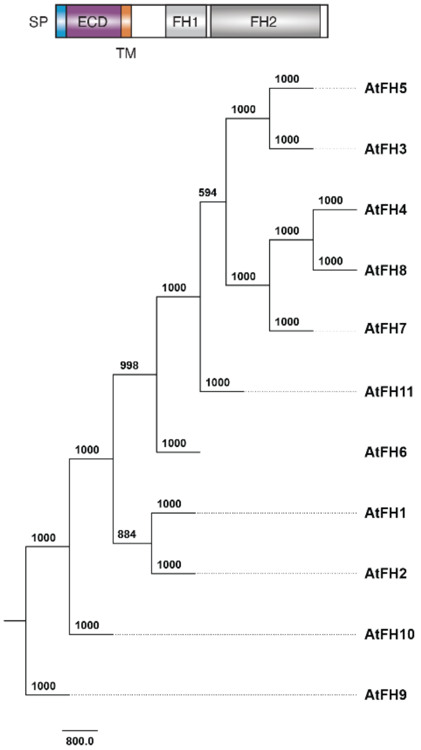


```
>AtFH5 ECD-234 aa
MVGMIRGGMGDQNWSRLVFWLILFSGLLVITLEENPEKDEIFLSQFMAPSTGQVNEHMEETSWA
QRCWQDSDCVKEAVAEFNLCFPGSKDSRELFGLNHTNLKQTLLDCIQEKGKLNGHNPKYLELLS
SMLDIPRRNLATKPGSSPSPSPSRPPKRSRGPPRPPTRPKSPPPRKSSFPPSRSPPPPPAKKNA
SKNSTSAPVSPAKKKEDHEKTIIIAVVVTAVSTFLLAALFFL

>AtFH3 ECD-174 aa
MGRLRLAFLAISLVVFVCVSEEIFSRGGLNLLRFSVYGEDVAEQTWIHQNPRRKLISYPKKFSV
SAPNLAFGPAPSFAPGPGPSFAPGPAPNPRSYDWLAPASSPNEPPAETPDESSPSPSEETPSVV
APSQSVPGPPRPPPQREKKDDILMKLIIAVASTAVLTFVFVALMFL

>AtFH4 ECD-100 aa
MAAMLMQPWPPFLPHLTLVFLTLILFFPNQSFSQSDSPRNIETFFPNDTITPPVQSPVLSPPQN
PSSSSSDSDRGNILRAVLITAASTLLVAAVFFFLVH

>AtFH8 ECD-98 aa
MAAMFNHPWPNLTLIYFFFIVVLPFQSLSQFDSPQNIETFFPISSLSPVPPPLLPPSSNPSPPS
NNSSSSDKKTITKAVLITAASTLLVAGVFFFCLQ

>AtFH11 ECD-178 aa
MVYFRQIFLMIIVVSLHCCKVRFFCIVANAKELDDWKVLTVENGERYRTHVGRYAGEEGGEKIK
LRVLEKFRALLDLIKPSTSRRRNLAESASFSPWPAPSPSPFFPNGGPIESPAYPPAPPRPIPPHL
RRPLPQRTHPLEQPEIQRRKHEKGGTFKKILVPVVASTASAIGFVVCVVG

>AtFH6 ECD-127 aa
MKALQSRFFFFFFFYIFFSVSVSSEAHRRILHQPLFPESSTPPPPDFQSTPSPPLPDTPDQPFF
PENPSTPQQTLFPPPPPPVSADVNGGLPIPTATTQSAKPGKKVAIVISVGIVTLGMLSALAFF

>AtFH1 ECD-128 aa
MLFFLFFFYLLLSSSSDLVFADRRVLHEPFFPIDSPPPSPPSPPPLPKLPFSSTTPPSSSDPNA
SPFFPLYPSSPPPPSPASFASFPANISSLIVPHATKSPPNSKKLLIVAISAVSSAALVALLIAL

>AtFH2 ECD-176 aa
MTTIPFCFLFVAFFFSSSTADQRHHSRHLLHQPFFPVVTAAPPPYQPPVSSQPPSPSPHTHHHH
KKHLTTTTPPPHEKHLFSSVANPPPPPPSPPHPNPFFPSSDPTSTASHPPPAPPPPASLPTFPA
NISSLLFPTHNKQSKPPSNGHIARLVTITASVISAAALLSLFAVFIIF

>AtFH10 ECD-122 aa
MDGLCYVIFIIFSLLSCAFSPLSYASPATFSRRHLLQAPVTDPSTFSPPFFPLYSSTSPPPPPS
PPQPLPPPAPTFATFPANISALVLPRSPKPQTPSRTLLIPAISAVLAAATLIALAFFF

>AtFH9 ECD-122 aa
MQNFWFAIFFFLLTCAPPSPLSYASTVTLSRRLLYDYESPLPLPLSPISPPFFPLESSPPSPPP
PLPPTPPTTFAVFPTFPANISALVLPRSSKPHHTSPTLLLPALSAVLVIATVIGLALF
```

EXT-like		AGP-like
$SP_{(2)}$	$SP_{(3\text{-}5)}$	$(XP)_n$
0	2	1
0	0	6
1	0	1
1	0	1
n/a	n/a	n/a
0	0	1
1	0	1
1	3	0
1	0	1
0	2	0
2	1	1

Figure S5. Maximum parsimony phylogeny of class I formins with 100 bootstrap replicates (bootstrap value indicated in nodes). On the left, the sequences of their respective ECDs and presumed glycomotifs. Amino acid residues in bold letters correspond to XP dipeptides where the letter X represents A,S,T or G. AGP-like motifs were annotated in the table only when at least two repeats were contiguous within the sequence. Bold, underlined amino acid residues correspond to EXT-like motifs. AtFH7 lacks a transmembrane domain. n/a: not applicable.

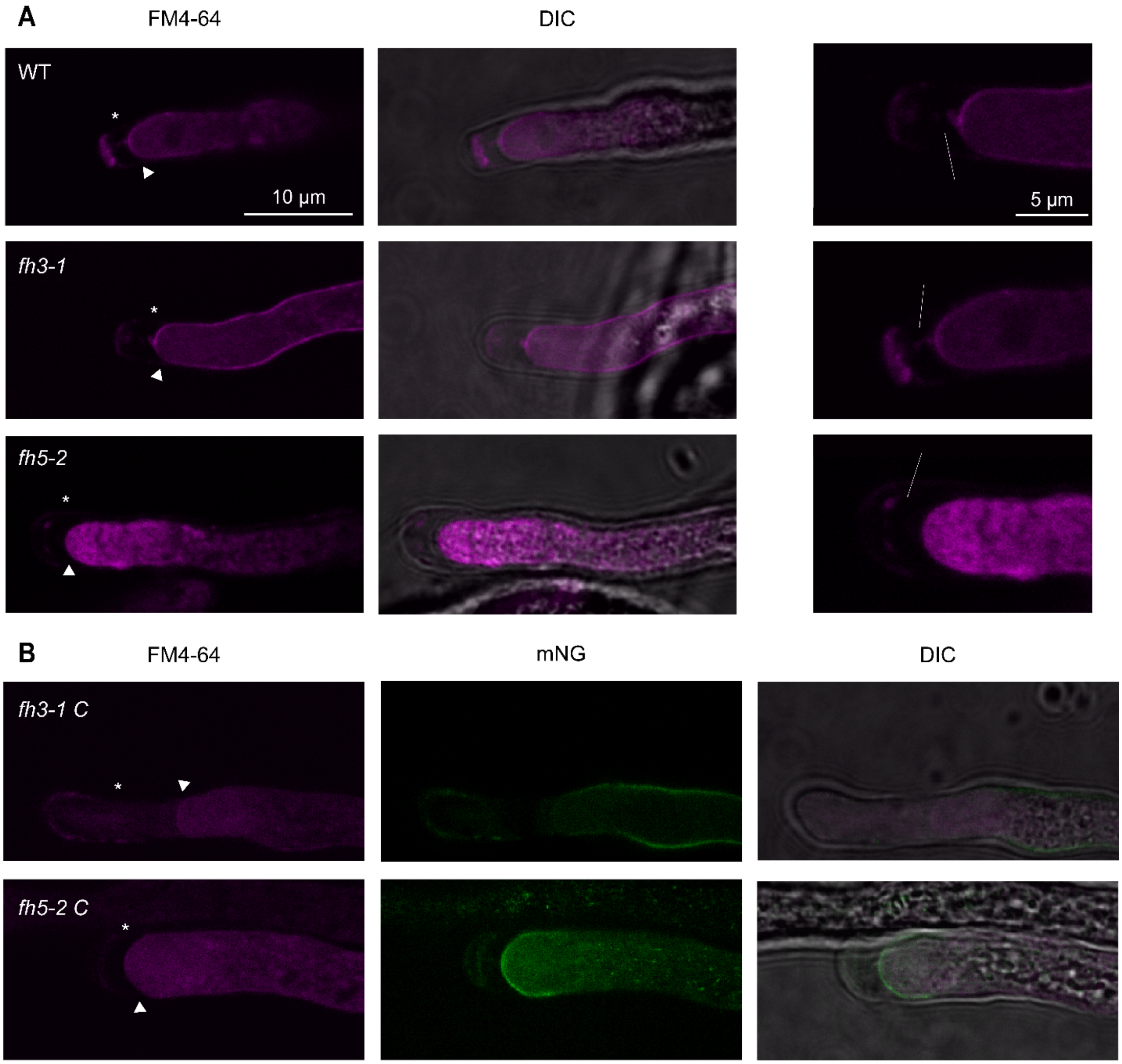

Figure S6. Wild type, *fh3-1* or *fh5-2* **(A)** pollen tubes, as well as lines complemented with the full length mNG translational fusions (*fh3-1 C* and *fh3-2 C*, **B**) were grown *in vitro* and then placed in hyperosmotic germination medium (25% sucrose) to induce plasmolysis and stained with FM4-64. '*' indicate the apoplastic space, white arrowhead indicates the retracted plasma membrane, arrows right panels in A, indicate membrane extensions (Hechtian strands).

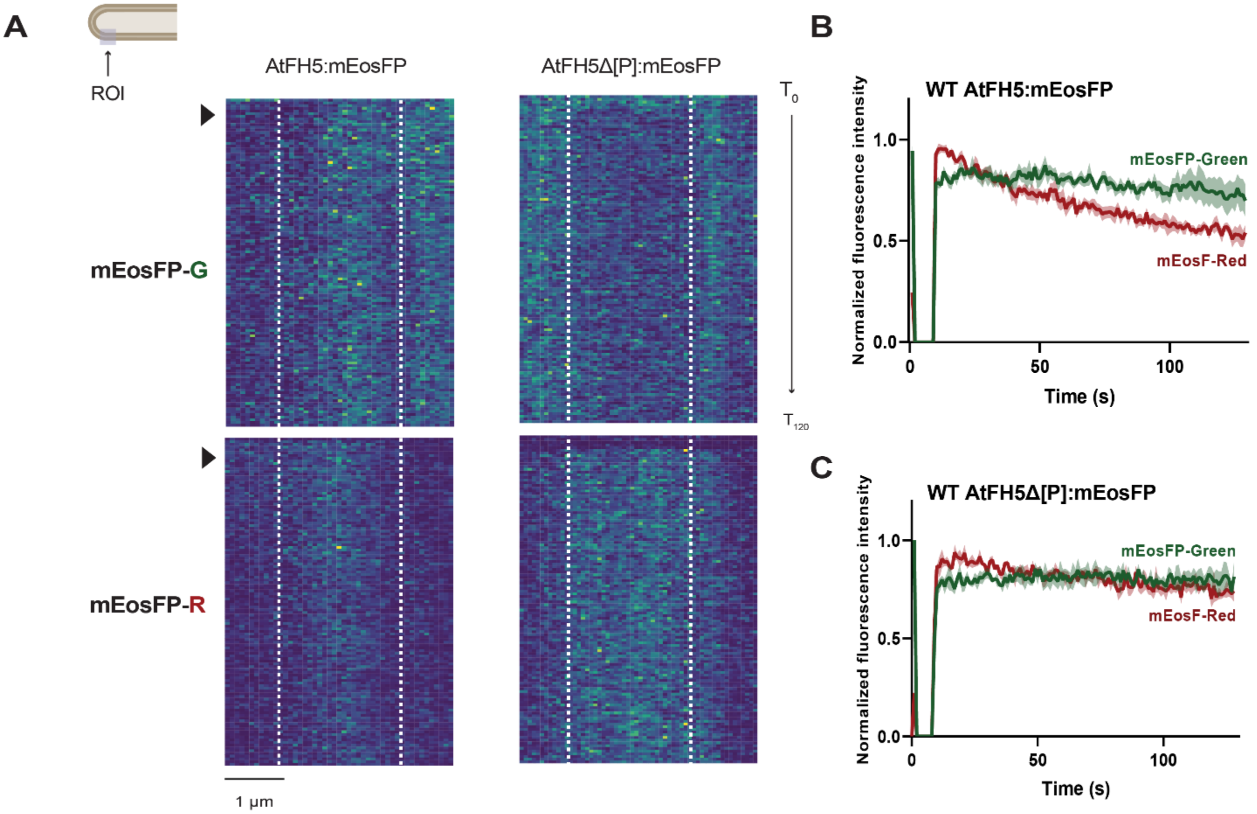

Figure S7. A) Lateral diffusion dynamics of AtFH5:mEosFP (left) or AtFH5Δ[P]:mEosFP (right) after photoconversion in the wild-type background. Kymographs represent the normalized fluorescence intensity in the photoconverted region (ROI indicated in pollen tube schematic corresponds to the region delineated with dashed white lines within kymographs) and surrounding area for the green form of mEosFP (mEosFP-G, top panels) or photoconverted red form of mEosFP (mEosFP-R, bottom panels) over time (T). Black arrowhead indicates the time of photoconversion. The color scale indicates the normalized fluorescence intensity from 0 to the highest intensity value possible, 1. **B)** Quantification of AtFH5 mean normalized fluorescence intensity (colored lines) of mEosFP-G or mEosFP-R in the ROI, pre and post photoconversion and standard error (shading), *n*=9. **C)** Quantification of AtFH5Δ[P]:mEosFP-G or AtFH5Δ[P]:mEosFP-R pre and post-photoconversion, *n*=8.

Table S1. Primers used in this study

Target	Purpose	Sequence 5' to 3'
AtFH3 CDS	Cloning	GGGGACAACTTTGTATACAAAAGTTGATGGGGAGATTGAGATTAGC
		GGGGACCACTTTGTACAAGAAAGCTGGGTACGAAGGTGAACTATCCTCTTC
AtFH3ΔECD	Overlap PCR/Cloning	TGTTTCCGAGCTTATCATCGCGGTTGCTTC
		CGATGATAAGCTCGGAAACACAAACGAAAAC
AtFH3Δ[P]	Overlap PCR/Cloning	CAACGGGAGAAGAAGGATGATATC
		ACCGAAAGCTAAATTTGGAGCA
AtFH5 CDS	Cloning	GGGGACAACTTTGTATACAAAAGTTGATGGTTGGAATGATTCGAGGAG
		GGGGACCACTTTGTACAAGAAAGCTGGGTATTAGTCTGAATCTGAACTAGACTGATC
AtFH5ΔECD	Overlap PCR/Cloning	TTTGGAAGAGATCATCATTGCTGTTGTTG
		CAATGATGATCTCTTCCAAAGTTATTACCAATAAC
AtFH5Δ[P]	Overlap PCR/Cloning	GCGAAAAAAAAAGAGGATCATGA
		GGAAGAGCCGGGTTTAGTAGC
AtFH5Δ{SPPP}	Overlap PCR/Cloning	GCTAAGAAAAACGCTTCTAAAAATTCAACT
		TTTTGGTCGAGTAGGTGGGC
AtFH1ecd	Cloning	GGGGACAAGTTTGTACAAAAAAGCAGGCTATGCTCTTCTTCTTATTCTTCTTC
		GGGGACCACTTTGTACAAGAAAGCTGGGTAGAGTAAAGCGATAAGTAGAGCG
AtFH3ecd	Cloning	GGGGACAAGTTTGTACAAAAAAGCAGGCTATGGGGAGATTGAGATTAGC
		GGGGACCACTTTGTACAAGAAAGCTGGGTAGCAACACAAGAACATCAATGC
AtFH5ecd	Cloning	GGGGACAAGTTTGTACAAAAAAGCAGGCTATGGTTGGAATGATTCGAGG
		GGGGACCACTTTGTACAAGAAAGCTGGGTATAAGAAGAATAATGCAGCCAA
fh3-1 (SALK_150350)	Genotyping	AAGAAGCTCTCGGAACTCTCG
		TCTTCACATCTCGCAAAATCC
fh5-2 (SALK_044464)	Genotyping	AGCGTTTTTCTTAGCAGGAGG
		TGGTTGATTCTGTTTTCTGGG
hpat1 (SALK_120066)	Genotyping	GTGATTATGATATGAAGGTAAGC
		AAATCTAGTGGAGACCAGAC
hpat2 (SM_3_38225)	Genotyping	ATTTCCAATCCCCATATTTGG
		CATTGTCACCAATGTCACCTG
hpat3 (SALK_04668)	Genotyping	AAGATACTGCAGTAAGGTCC
		GACAAGAAGGGAAGTAAAGG
SALK LB1.3	Genotyping	ATTTTGCCGATTTCGGAAC
SPM32 LB	Genotyping	TACGAATAAGAGCGTCCATTTTAGAGTGA

Table S2. Likelihood-ratio test (model fit parameters) and modeling output

**Model (lme4 syntax)**	χ^2	**p-value**
y = MFI ~ group*distance + (1|cell)	16748	<0.001
**Random Effects**	**Variance**	**SD**
**Residual**	621.73	24.93
**Random effects (cell)**	1229.04	35.06
**Number of cells**	99	
**Number of observations**	20,988	
**Marginal R^2 / Conditional R^2**	0.313 / 0.769	

MFI = mean fluorescence intensity

Distance = distance in μm from the tip

Group = combination of genotype-construct (WT AtFH5:mNG, WT AtFH5Δ[P]:mNG, WT AtFH5Δ{SPPP}:mNG and *hpat1,2,3* AtFH5:mNG, *hpat1,2,3* AtFH5Δ[P]:mNG, *hpat1,2,3* AtFH5Δ{SPPP}:mNG).
